# Functional Tumor Targeting Nano‐Systems for Reprogramming Circulating Tumor Cells with In Situ Evaluation on Therapeutic Efficiency at the Single‐Cell Level

**DOI:** 10.1002/advs.202105806

**Published:** 2022-05-20

**Authors:** Xiao‐He Ren, Xiao‐Yan He, Chang Xu, Di Han, Si‐Xue Cheng

**Affiliations:** ^1^ Key Laboratory of Biomedical Polymers of Ministry of Education Department of Chemistry Wuhan University Wuhan 430072 P. R. China; ^2^ School of Life Sciences Anhui Medical University Hefei 230032 P. R. China

**Keywords:** circulating tumor cells, CRISPR‐Cas9, gene therapy, molecular beacons, targeting delivery

## Abstract

Tumor heterogeneity is primarily responsible for treatment resistance and cancer relapses. Being critically important to address this issue, the timely evaluation of the appropriateness of therapeutic actions at the single‐cell level is still facing challenges. By using multi‐functionalized nano‐systems with the delivery vector composed of histone for plasmids loading, hyaluronic acid for tumor targeting, and a fusion peptide for C‐X‐C motif chemokine receptor 4 (CXCR4） targeting as well as nuclear localization, the reprogramming of circulating tumor cells (CTCs) with in situ detection on biomarkers at the single‐cell level is realized. By efficient co‐delivery of the genome editing plasmid for CXCR4 knockout and molecular beacons for detection of upregulated mRNA biomarkers into CTCs in unprocessed whole blood, the therapeutic outcomes of genome editing at the single‐cell level can be in situ evaluated. The single‐cell analysis shows that CXCR4 in CTCs of cancer patients is efficiently downregulated, resulting in upregulated anticancer biomarkers such as p53 and p21. The study provides a facile strategy for in‐depth profiling of cancer cell responses to therapeutic actions at single‐cell resolution to evaluate the outcomes of treatments timely and conveniently.

## Graphical abstract

Using multi‐functionalized nano‐systems to co‐deliver the genome editing plasmid and molecular beacons, the therapeutic outcomes of genome editing on circulating tumor cells of cancer patients can be directly visualized. This study provides a facile strategy for in‐depth profiling of cancer cell responses to therapeutic actions at single‐cell resolution to evaluate the outcomes of treatments timely and conveniently.

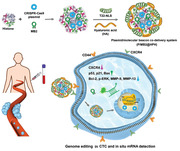

## 1 Introduction

As a fundamental property of cancers, cancer heterogeneity resulting from genetic, epigenetic, and/or phenotypic changes leads to different levels of sensitivity to cancer therapies and provides the fuel for the development of therapy resistance, which becomes a critical hurdle in cancer therapy.^[^ ^1^, ^2^ ^]^ Timely and accurate assessments of the feasibility and appropriateness of particular therapeutic actions on heterogeneous cancer cells are of crucial importance to develop more effective personalized therapies. However, analysis of the primary tumor and metastatic lesions by multiple and repeated tissue biopsies is not clinically feasible. An alternative strategy, analysis of circulating tumor cells (CTCs) as a noninvasive liquid biopsy technique provides unique insights into tumor heterogeneity by studying molecular features of cancers at single‐cell resolution.^[^ ^3^, ^4^, ^5^ ^]^ Besides, CTCs play a crucial role in cancer metastasis.^[^ ^3^ ^]^ Single‐cell profiling of CTCs can unravel real‐time responses to cancer treatment and evaluate metastatic risk.^[^ ^5^ ^]^

As it is well known, genomic changes play a curial role in tumorigenesis and neoplastic progression. Genome editing is a robust strategy for cancer treatment and cancer research.^[^ ^6^ ^]^ However, the safety and efficacy are critical concerns of genome editing, especially for in vivo studies. Enjoying safety benefits, the ex vivo editing approach not only allows therapeutic actions but also provides a facile platform for exploring cancer gene therapy.^[^ ^7^, ^8^ ^]^ Most commonly, ex vivo editing involves complicated procedures (e.g., cell collection, isolation, and editing). As far as we know, ex vivo genome editing on CTCs in whole blood has never been reported.

The purpose of this study is to develop a platform for ex vivo genome editing on CTCs with in situ evaluation of therapeutic efficiency at the single‐cell level to unravel real‐time responses to cancer treatments for personalized cancer therapy. By using a highly efficient delivery vector based on natural occurring biomacromolecules to specifically deliver the CRISPR‐Cas9 plasmid and molecular beacons to CTCs in peripheral blood of cancer patients, the therapeutic interventions of genome editing can be assessed in situ by detection of multiple mRNAs including mRNA of the targeting protein and other mRNAs playing important roles in cancer progression. The genome editing was directly carried out in CTCs in whole blood without isolation to mimic the in vivo process as well as to eliminate the unfavorable effects of cell isolation on CTC activity.

As far as we know, the co‐delivery systems for simultaneous gene editing and mRNA probing in CTCs have never been reported. Our investigation provides a facile ex vivo strategy for evaluating the efficiency of therapeutic actions in a particular patient to provide accurate information for personalized therapy by using a few milliliters of peripheral blood containing CTCs.

In this study, C‐X‐C motif chemokine receptor 4 (CXCR4), a typical target protein in cancer therapy, was selected as a representative target of ex vivo genome editing. As a promising therapeutic target and an important prognostic cancer biomarker, CXCR4 plays a vital role in the crosstalk between cancer cells and the microenvironment for cancer development.^[^ ^9^, ^10^, ^11^, ^12^ ^]^ For example, the CXCR4/CXCL12 axis promotes tumor progression and metastasis by regulating the MAPK and PI3K‐AKT pathways.^[^ ^13^ ^]^ In addition, the activation of CXCR4 promotes epithelial‐to‐mesenchymal transition (EMT) and increases the secretion of matrix metalloproteinases (MMPs) that facilitate the process of invasion.^[^ ^14^ ^]^ Inhibition of CXCR4 by CXCR4 antagonists,^[^ ^15^, ^16^, ^17^ ^]^ CXCR4 siRNA,^[^ ^18^, ^19^ ^]^ and CRISPR‐based genome editing^[^ ^20^, ^21^, ^22^, ^23^, ^24^ ^]^ is a promising therapy strategy in the treatment of a variety of cancers.

Since viral vehicles suffer from susceptibility to mutagenesis, carcinogenesis, and immunogenicity,^[^ ^25^ ^]^ various non‐viral vectors have been developed for delivering genome editing systems to cancer cells.^[^ ^26^, ^27^, ^28^, ^29^, ^30^, ^31^, ^32^, ^33^ ^]^ However, as far as we know, there are no reports on the vectors for delivery of genome editing systems to CTCs in whole blood. Besides, among diverse non‐viral vectors, such as cationic lipids, cationic synthetic polymers, inorganic nanocarriers (e.g., gold nanoparticles, and mesoporous silicon nanoparticles), our biomacromolecule based vectors exhibit ideal biocompatibility, which minimizes the unfavorable effects, such as perturbing microRNA levels in treated cells, to ensure the accurate detection on therapeutic responses.^[^ ^34^ ^]^

## 2 Results and Discussion

**Figure 1:**
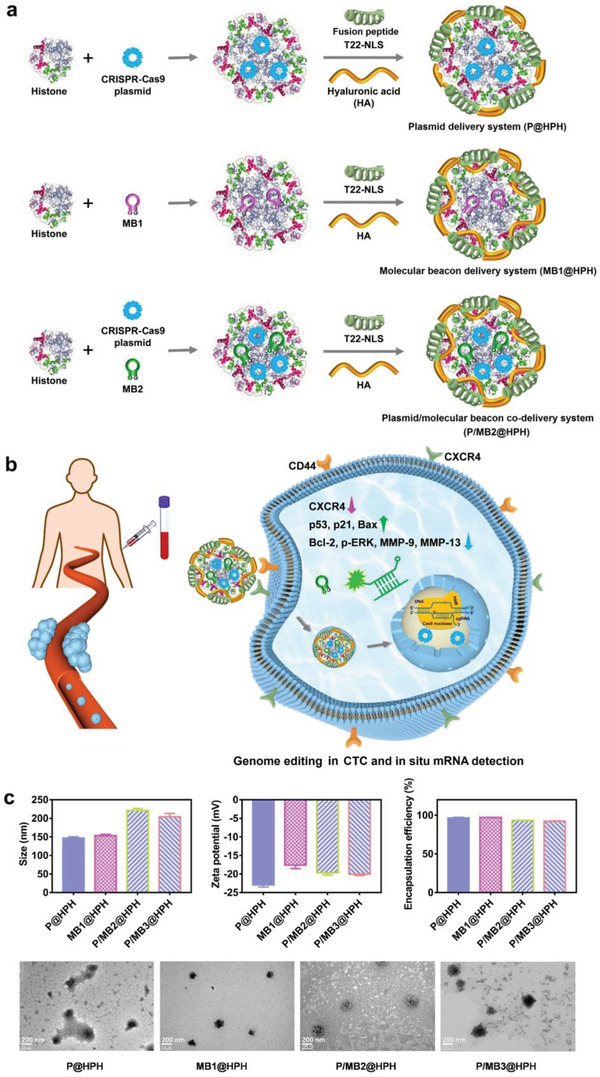
Plasmid and molecular beacon delivery systems for reprogramming CTCs and in situ evaluation on therapeutic efficiency for personalized cancer therapy. a) Schematic structures of cancer‐targeting plasmid and molecular beacon delivery systems decorated by a fusion peptide (T22‐NLS) and hyaluronic acid (HA). b) Schematic diagram showing CXCR4 knockout and in situ probing of mRNA to evaluate therapeutic efficiency in a CTC by a plasmid/molecular beacon co‐delivery system. c) The size, zeta potential, plasmid/molecular beacon encapsulation efficiency, and TEM images of cancer‐targeting plasmid and molecular beacon delivery systems. Data are mean ± s.d, n = 3.

To realize highly efficient genome editing with in situ evaluation on therapeutic efficiency, a series of nano‐systems (plasmid delivery systems for genome editing, molecular beacon delivery systems for detection of downregulated CXCR4 mRNA, and plasmid/molecular beacon co‐delivery systems for genome editing and detection of upregulated p53 mRNA and p21 mRNA, as detailed in Figure S1, Supporting Information) were constructed. The structures of representative delivery systems are shown in **Figure** **1**a. To realize an efficient cellular delivery, histone complexes with plasmids and/or molecular beacons form the complexed cores through electrostatic interactions. Afterwards, negatively charged hyaluronic acid (HA) complexed with T22‐NLS fusion peptide is self‐assembled onto the positively charged complexed cores. Positively charged histone has inherent DNA condensation capabilities. Besides, nuclear localization signal (NLS) sequences in histone facilitate nucleus entry.^[^ ^35^ ^]^ The negatively charged HA can target CD44 receptors overexpressed on cancer cells,^[^ ^36^ ^]^ enhancing the identification of neoplastic cells. In addition, the degradation of HA by hyaluronidase triggers a charge reversion promoting the endosomal escape.^[^ ^37^ ^]^ The fusion peptides, T22‐NLS can specifically combine with CXCR4 overexpressed on cancer cells to promote cellular uptake by the T22 sequence,^[^ ^38^, ^39^ ^]^ and enhance nucleus translocation by the NLS sequence.^[^ ^40^ ^]^ With the combination of these functional components, the multifunctional delivery vector can specifically deliver the geneediting plasmid to cell nuclei to effectively mediate CXCR4 knockout. The downregulated CXCR4 in cancer cells results in inhibited growth, migration, and invasion with downregulated Bcl‐2, p‐ERK, MMP‐9, and MMP‐13 and upregulated p53, p21, and Bax. By co‐delivery of a molecular beacon with the genome editing plasmid, the upregulated mRNA of a particular protein (e.g., p53) can be in situ probed (Figure 1b). To avoid interferences from hybridization with particular mRNAs before the complete genome editing, the downregulated biomarkers (e.g., CXCR4 mRNA) have to be detected by molecular beacon delivery systems after genome editing. The properties of genome editing plasmid and/or molecular beacon delivery systems are detailed in Figure 1c.

To achieve efficient genome editing, three different sgRNA sequences in CRISPR‐Cas9 plasmids were compared, and sgRNA_3_ was identified as the most effective one for CXCR4 knockout (Figure S2, Supporting Information). Therefore, sgRNA_3_ was used to construct the CRISPR‐Cas9 plasmid for further studies.

To study the effects of the functional components in the cancer‐targeting delivery, the CRISPR‐Cas9 plasmid was loaded in vectors with different compositions. All plasmid‐loaded nanoparticles have sizes within 200 nm. The size increases after the decoration of T22‐NLS peptide and/or HA. Besides, after decoration of negatively charged HA and peptide/HA complexes onto the positively charged CRISPR‐Cas9 plasmid/histone complexes (P@H), the resultant CRISPR‐Cas9 plasmid@histone/HA (P@HH) and CRISPR‐Cas9 plasmid@histone/peptide/HA (P@HPH) exhibit negative zeta potentials. All nanoparticles show satisfactory plasmid encapsulation efficiencies higher than 85% (Figure S1a, Supporting Information).

**Figure 2:**
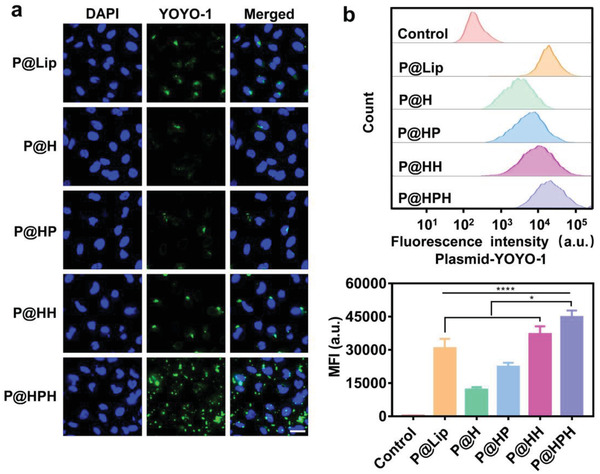
Study on cellular internalization of plasmid delivery systems in cancer cells. a) CLSM images of HeLa cells treated by different plasmid delivery systems. CRISPR‐Cas9 plasmid was labeled by YOYO‐1 (green), and cell nuclei were stained by DAPI (blue). Scale bar: 36 µm. b) Flow cytometry analysis on HeLa cells treated by different plasmid delivery systems. HeLa cells were co‐incubated with plasmid delivery systems for 4 h. Untreated HeLa cells were served as a control. Data are mean ± s.d, n = 3. Statistical analysis was performed by using one‐way analysis of variance (ANOVA) with Tukey's multiple comparison test. *P< 0.05, ****P< 0.0001.

To assess cellular uptake, HeLa cells incubated with different nanoparticles loaded with the YOYO‐1 labeled CRISPR‐Cas9 plasmid were visualized by confocal laser scanning microscopy (CLSM) and quantitated by flow cytometry (**Figure** **2**). The cellular uptake of P@HP and P@HH nanoparticles is higher than that of P@H because of the introduction of T22‐NLS and HA, respectively. P@HPH nanoparticles possess the most significantly enhanced cell uptake due to the targeting capability of T22 sequence and HA for CXCR4 and CD44, respectively, overexpressed on malignant HeLa cells (Figure S3, Supporting Information). The endosomal escape of P@HPH nanoparticles in HeLa cells was observed by CLSM (Figure S4, Supporting Information). The yellow co‐localization dots formed by YOYO‐1 labeled plasmid and LysoTracker stained lyso/endosomes were observed after 2 h incubation, which gradually disappeared after 4 h incubation owing to the lyso/endosomal escape.

For noncancerous HEK293 cells, although T22‐NLS and/or HA decoration increases the cellular uptake slightly, no statistically significant difference exists between different delivery systems (Figure S5, Supporting Information) since neither CD44 nor CXCR4 is overexpressed in 293 cells.

**Figure 3:**
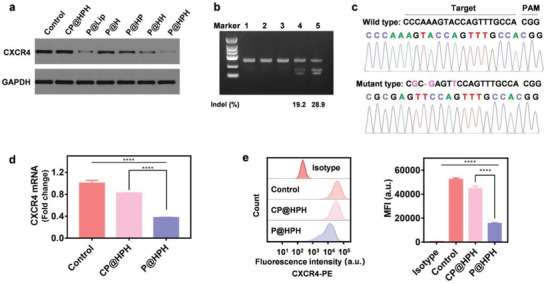
Study on genome editing in cancer cells. a) Western blot analysis on CXCR4 expression in HeLa cells after different treatments. b) Surveyor assay on the genomic DNA extracted from HeLa cells after different treatments (1) untreated control, 2) P@Lip, without DNA denaturation and rehybridization, 3) P@HPH, without DNA denaturation and rehybridization, 4) P@Lip, with DNA denaturation and rehybridization, and 5) P@HPH, with DNA denaturation and rehybridization). c) The DNA sequencing result of edited HeLa cells treated by the targeting CRISPR‐Cas9 plasmid delivery system (P@HPH) as compared with untreated cells. d) qPCR assay on CXCR4 mRNA in HeLa cells after different treatments. e) Flow cytometry analysis on antibody labeled CXCR4 protein in HeLa cells after different treatments. HeLa cells were treated by plasmid delivery systems for 48 h. Untreated cells were served as a control. Data are mean ± s.d, n = 3. Statistical analysis was performed by using one‐way analysis of variance (ANOVA) with Tukey's multiple comparison test. ****P< 0.0001.

Further, the genome editing efficiencies of different plasmid delivery systems were compared in neoplastic cells. All CRISPR‐Cas9 plasmid‐loaded nanoparticles can downregulate CXCR4 expression as determined by Western blotting. Being consistent with the cellular uptake study, HA and/or T22‐NLS modification improves the genome editing efficiency, and P@HPH with both HA and T22‐NLS results in the most effective CXCR4 knockout in cancer cells (**Figure** **3**a). T7E1 assay indicates the genome editing efficiency of multiple cancer‐targeting delivery systems, P@HPH, is much higher than that of P@Lip with the commercial gene vector (Figure 3b). The DNA sequencing confirms the mutations after genome editing by P@HPH (Figure 3c).

qPCR confirms CXCR4 mRNA in genome‐edited cells treated by P@HPH loaded with the CRISPR‐Cas9 plasmid dramatically reduces as compared with unedited cells treated by CP@HPH loaded with the control plasmid (Figure 3d). Flow cytometry analysis on fluorescent antibody labeled CXCR4 verifies the significantly downregulated CXCR4 on the cell surface of the edited HeLa cells (Figure 3e). Clearly, the above results confirm P@HPH nanoparticles can efficiently exert genome editing to knock out CXCR4.

**Figure 4:**
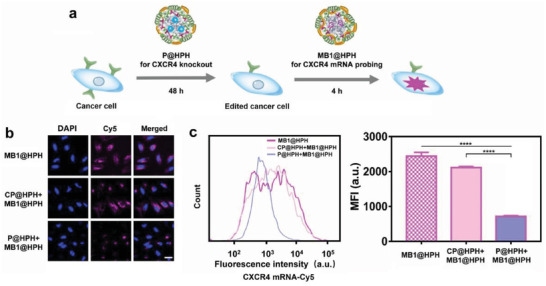
Detection of CXCR4 mRNA in genome‐edited and unedited cancer cells by the cancer‐targeting molecular beacon delivery system. a) The procedure of genome editing and CXCR4 mRNA detection. b) CLSM observation on CXCR4 mRNA probed by the molecular beacon delivery system in HeLa cells after different treatments. Cell nuclei were stained by DAPI (blue). Scale bar: 36 µm. c) Flow cytometry analysis on CXCR4 mRNA in HeLa cells after different treatments. Data are mean ± s.d, n = 3. Statistical analysis was performed by using one‐way analysis of variance (ANOVA) with Tukey's multiple comparison test. ****P< 0.0001.

In our investigation, the CXCR4 knockout was further studied by the detection of intracellular CXCR4 mRNA using a molecular beacon delivery system. After genome editing by P@HPH for 48 h, the edited cells were co‐incubated with MB1@HPH to allow intracellular mRNA detection by hybridization of MB1 and CXCR4 mRNA to restore the fluorescence (**Figure** **4**a). The edited cells exhibit dramatically reduced CXCR4 mRNA as visualized by CLSM (Figure 4b) and quantitated by flow cytometry (Figure 4c).

After CXCR4 knockout, the growth inhibition in edited cancer cells can be observed, and P@HPH with cancer‐targeting capability mediated by HA/CD44 and T22/CXCR4 interactions results in the strongest inhibition in cancer cells as studied by MTT assay. As expected, control plasmid‐loaded delivery systems do not affect the cell growth. After being treated by different plasmid delivery systems, the proliferation of both edited and unedited noncancerous 293 cells does not change obviously since the native 293 cells do not overexpress CXCR4; thus the CXCR4 knockout would not apparently affect the growth of 293 cells (Figure S6, Supporting Information). Besides, the delivery systems do not target 293 cells and cellular uptake of the plasmid delivery systems is limited.

The flow cytometry analysis on the HeLa cells stained with Annexin V‐FITC and PI shows unedited cells treated with CP@HPH do not exhibit increased cell apoptosis as compared with untreated cells. While the edited cells treated with P@HPH show obviously increased apoptosis (Figure S7a, Supporting Information), which is in coincidence with previous reports that indicate CXCR4 inhibition results in the apoptosis of cancer cells.^[^ ^41^, ^42^ ^]^

According to Western blot analysis, as compared with unedited cells, genome‐edited cancer cells exhibit significantly upregulated p53, p21, and Bax as well as downregulated Bcl‐2 (Figure S7b, Supporting Information). Consistently, qPCR analysis confirms the upregulation of mRNA levels of p53 and p21 (Figure S7c, Supporting Information). Besides, qPCR further indicates the genome‐edited cancer cells have apparently reduced microRNA‐21 and microRNA‐221 (Figure S8, Supporting Information). Based on literatures, p53 can regulate cell apoptosis precisely by Bcl‐2 family such as Bax.^[^ ^43^ ^]^ Inhibition of anti‐apoptotic microRNAs, such as microRNA‐21 and microRNA‐221, enhances the p53‐mediated expression of pro‐apoptotic microRNAs to induce apoptosis.^[^ ^44^ ^]^ p21 plays a vital role in promoting anti‐proliferative activities.^[^ ^45^ ^]^ CXCR4 inhibition leads to reduced cell proliferation and growth, and induces cell cycle arrest and apoptosis.^[^ ^23^, ^41^, ^42^ ^]^ Our results are in good agreement with previous studies.

**Figure 5:**
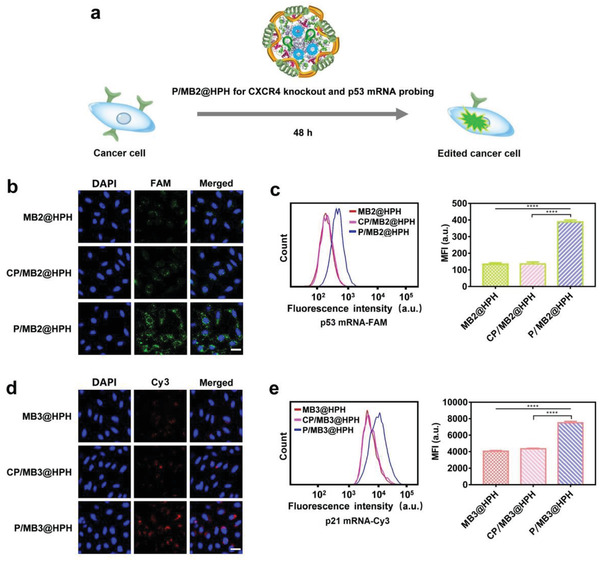
Detection of p53 mRNA and p21 mRNA in genome‐edited and unedited cancer cells by cancer‐targeting plasmid/molecular beacon co‐delivery systems. a) The procedure of genome editing and p53 mRNA detection by the co‐delivery system. b) CLSM observation on p53 mRNA probed by the plasmid/molecular beacon co‐delivery system in HeLa cells after different treatments. Cell nuclei were stained by DAPI (blue). Scale bar: 36 µm. c) Flow cytometry analysis on p53 mRNA in HeLa cells after different treatments. d) CLSM observation on p21 mRNA probed by the plasmid/molecular beacon co‐delivery system in HeLa cells after different treatments. Cell nuclei were stained by DAPI (blue). Scale bar: 36 µm. e) Flow cytometry analysis on p21 mRNA in HeLa cells after different treatments. The cells treated by molecular beacon delivery systems were also studied for comparison. Data are mean ± s.d, n = 3. Statistical analysis was performed by using one‐way analysis of variance (ANOVA) with Tukey's multiple comparison test. ****P< 0.0001.

Due to the vital role of p53 and p21 in cancer prevention, the intracellular p53 mRNA and p21 mRNA in cancer cells were in situ probed by plasmid/molecular beacon co‐delivery systems (P/MB2@HPH for CXCR4 knockout and p53 mRNA detection, and P/MB3@HPH for CXCR4 knockout and p21 mRNA detection). Both CLSM and flow cytometry show the fluorescence intensity induced by hybridization of the molecular beacon with p53 mRNA or p21 mRNA in edited cancer cells greatly increases as compared with that in unedited cells (**Figure** **5**).

The important characteristics of cancer metastasis and recurrence are the capabilities of migration and invasion. In this study, the effects of genome editing on the proteins involved in metastasis and invasion were studied. As revealed by Western blot assay, phosphorylated ERK (p‐ERK) for regulating cell motility, snail, vimentin, and twist for promoting EMT, as well as MMP‐9 and MMP‐13 for degrading extracellular matrix are greatly downregulated in edited cells after CXCR4 knockout. Besides, E‐cadherin for maintaining cell–cell interactions are upregulated (Figure S9, Supporting Information). Clearly, CXCR4 knockout has favorable effects on preventing cancer metastasis and invasion.

Further, the cancer cell migration was evaluated via the wound healing assay and cell invasion was studied via the transwell assay. Compared with untreated HeLa cells (control), the edited cells treated with P@HPH exhibit dramatically suppressed migration and invasion, that is, the number of migrated cells decreases by ≈60% and the number of invaded cells decreases by more than 80% after CXCR knockout (Figure S10, Supporting Information).

According to the literatures, CXCR4 promotes cancer migration and invasion by inducing the expression of MMP‐9 and MMP‐13 via the ERK signaling pathway.^[^ ^46^ ^]^ Overexpressed CXCR4 promotes EMT.^[^ ^47^, ^48^, ^49^, ^50^ ^]^ Inhibition of CXCR4 could suppress cancer migration and invasion.^[^ ^22^, ^23^ ^]^ Our results are in accordance with the previous reports.

After confirming the robust effects of CXCR4 knockout on regulating cell behaviors of cancer cells, we further exerted genome editing on CTCs from cancer patients (see Table S1, Supporting Information, for clinicopathological information of cancer patients).

To confirm our delivery system is stable in whole blood, the targeting delivery system P@HPH prepared by FITC labeled T22‐NLS and TOTO‐3 labeled genome editing plasmid was directly added to the whole blood containing CTCs. After co‐incubation for 4 h, CTCs were isolated and observed by CLSM (Figure S11, Supporting Information). The fluorescence signals from FITC labeled T22‐NLS peptide and TOTO‐3 labeled plasmid are clearly observed in CTCs, indicating both the peptide and the plasmid enter CTCs in the form of nanoparticles and the nanoparticles are stable in whole blood. Besides, the genome editing plasmid can be efficiently delivered to all CTCs.

**Figure 6:**
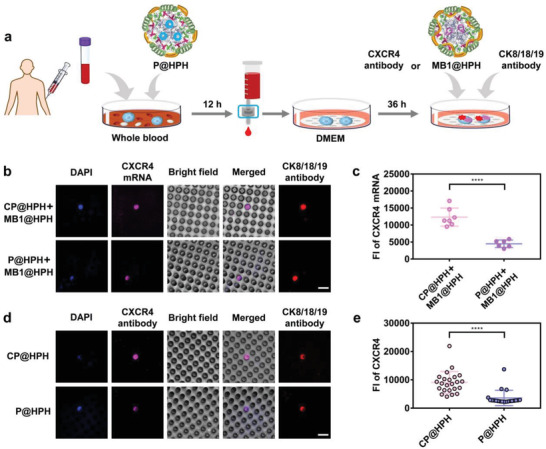
Genome editing on CTCs by the cancer‐targeting delivery system and detection on CXCR4 in genome‐edited CTCs as compared with unedited CTCs. a) The procedure of genome editing, and detection on CXCR4 mRNA and CXCR4. b) CLSM observation on unedited and edited CTCs with CXCR4 mRNA probed by the molecular beacon delivery system. Cell nuclei were stained by DAPI (blue). Scale bar: 15 µm. c) Fluorescence intensity of CXCR4 mRNA probed by the molecular beacon delivery system in unedited and edited CTCs. All CTCs from the patient BC1 are shown in Figure S13, Supporting Information. Fluorescence intensity of each CTC was analyzed by Volocity Demo 6.1.1 software. d) CLSM observation on unedited and edited CTCs with antibody labeled CXCR4 protein. Cell nuclei were stained by DAPI (blue). Scale bar: 15 µm. e) Fluorescence intensity of antibody labeled CXCR4 protein in unedited and edited CTCs from the patient SCLC. Fluorescence intensity of each CTC was analyzed by Volocity Demo 6.1.1 software. Statistical analysis was performed by using Student's t‐test. ****P< 0.0001.

To study the effects of CXCR4 knockout on CTC behaviors and compare the behaviors of edited CTCs with unedited CTCs, the cancer‐targeting CRISPR plasmid delivery system (P@HPH) and the control plasmid delivery system (CP@HPH) were directly added into 2 mL of unprocessed whole blood from cancer patients, respectively. After 12 h, to avoid the unfavorable effects due to the viscosity enhancement of the whole blood, CTCs were isolated from the whole blood and the CTCs on the filter membrane were further incubated in DMEM for 36 h (**Figure** **6**a). The calcein‐AM viability assay confirms CTCs are alive after genome editing by P@HPH for 48 h (Figure S12, Supporting Information).

After genome editing, to detect the level of CXCR4 mRNA, the molecular beacon delivery system with the same targeting vector, MB1@HPH, was added to CTCs, followed by incubation for 4 h to allow the delivery of MB1 into CTCs for hybridization with CXCR4 mRNA. For comparison, CXCR4 protein on the surface of CTCs was labeled by the CXCR4 antibody. CLSM observation shows the edited CTC has a weaker fluorescence generated from the hybridization of MB1 and CXCR4 mRNA as compared with the unedited CTC (Figure 6b). The comparison of the fluorescence intensities of individual edited and unedited cells indicates the level of CXCR4 mRNA significantly decreases after genome editing (Figure 6c, CTCs shown in Figure S13, Supporting Information). The antibody labeling verifies the same trend, that is, after CXCR4 knockout by P@HPH, CXCR4 protein on the edited CTC surface decreases considerably (Figure 6d,e). These results confirm P@HPH can efficiently deliver CRISPR‐Cas9 plasmid into CTCs in unprocessed whole blood to realize the successful CXCR4 knockout. In addition, the cells were also labeled by CK8/18/19 antibodies. The overlap of fluorescence signals induced by CK8/18/19 and CXCR4 mRNA (or CXCR4 protein) in the same cells confirms the cells we detected are CTCs.

**Figure 7:**
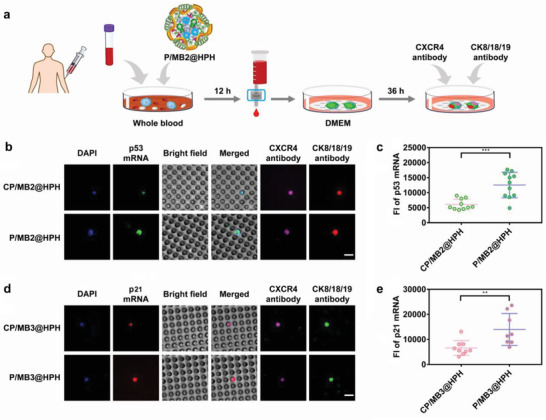
Genome editing on CTCs and detection on p53 and p21 in genome‐edited CTCs as compared with unedited CTCs by cancer‐targeting plasmid/molecular beacon co‐delivery systems. a) The procedure of genome editing and p53 mRNA detection. b) CLSM observation on unedited and edited CTCs with p53 mRNA probed by plasmid/molecular beacon co‐delivery systems. Cell nuclei were stained by DAPI (blue). Scale bar: 15 µm. c) Fluorescence intensity of p53 mRNA in unedited and edited CTCs probed by plasmid/molecular beacon co‐delivery systems. All CTCs from the patient BC2 are shown in Figure S14, Supporting Information. Fluorescence intensity of each CTC was analyzed by Volocity Demo 6.1.1 software. d) CLSM observation on unedited and edited CTCs with p21 mRNA probed by plasmid/molecular beacon co‐delivery systems. Cell nuclei were stained by DAPI (blue). Scale bar: 15 µm. e) Fluorescence intensity of p21 mRNA in unedited and edited CTCs probed by plasmid/molecular beacon co‐delivery systems. All CTCs from the patient BC3 are shown in Figure S15, Supporting Information. Fluorescence intensity of each CTC was analyzed by Volocity Demo 6.1.1 software. Statistical analysis was performed by using Student's t‐test. **P< 0.01, ***P< 0.001.

For the proteins upregulated after genome editing, the molecular beacons for their mRNA detection can be co‐loaded with the genome editing plasmid to realize in situ evaluation in the reprogrammed CTCs. Herein, the CRISPR plasmid/molecular beacon co‐delivery system was added to 2 mL of whole blood of patients for CXCR4 knockout and in situ probing of unregulated mRNA as detailed in **Figure** **7**a. The CLSM visualization demonstrates p53 mRNA and p21 mRNA are significantly upregulated in edited CTCs (Figure 7b–e). The fluorescence intensities induced by p53 mRNA (Figure 7c, CTCs shown in Figure S14, Supporting Information) and p21 mRNA (Figure 7e, CTCs shown in Figure S15, Supporting Information) in all edited and unedited CTCs were measured by the attendant software of CLSM. The mean fluorescence intensities of green fluorescence induced by p53 mRNA and red fluorescence induced by p21 mRNA in edited CTCs are approximately twofold higher than that of unedited CTCs. Besides, we also confirm the successful CXCR4 knockout in the CTCs for p53 mRNA and p21 mRNA detection by antibody labeling (Figure S16, Supporting Information).

Herein, the molecular beacons probing p53 mRNA and p21 mRNA were designed based on the sequences of wild‐type p53 and p21. It should be noted that mRNAs of non‐mutated and mutated p53 and p21 may co‐exist in the samples from patients. It is possible that MBs may hybridize with some types of mRNAs of mutated p53 and mutated p21. Whether the hybridization occurs or not is dependent on the position of the mutations, which may be different for different patients. Theoretically, if we identify the detailed position of the mutations for each particular patient, we can optimize the design of MB sequences to ensure that MBs only hybridize with wild‐type mRNAs of p53 and p21. This is an area requiring further study.

As detailed above, the multiple functional delivery systems can realize efficient genome editing and mRNA detection on CTCs from cancer patients. As well known, high expression levels of CXCR4 are predictive of poor prognosis in cancer patients.^[^ ^51^ ^]^ The above results imply that our genome editing systems may prevent cancer progression and metastasis by effectively reprograming CTCs to reverse oncogenic properties.

By using the multiple functional CTC targeting delivery vectors we developed, both therapeutic and diagnostic agents can be delivered into living CTCs in peripheral blood efficiently to exert ex vivo anti‐cancer treatments with in situ detection on therapeutic responses of particular patients. Owing to the superiorities of noninvasive blood biopsies such as easy accessibility for frequent detections, our approach provides an avenue to conveniently tailor and optimize therapeutic strategies according to the genomic characterization of CTCs and the therapeutic responses at the single‐cell level for each patient during the anticancer therapy.

## 3 Conclusion

In summary, by using multiple functional CTC targeting delivery systems, we realize robust genome editing in CTCs in whole blood from cancer patients. More importantly, the therapeutic efficiency can be in situ evaluated to provide important information for personalized cancer therapy. Due to the enhanced cellular uptake mediated by T22 targeting CXCR4 and HA targeting CD44 in malignant cells, our delivery vector can target cancer cells as well as CTCs in whole blood to realize efficient genome editing and mRNA detection. The genome editing delivery system exerts anti‐cancer effects through downregulation of proteins promoting cancer progression and upregulation of anticancer proteins such as p53 and p21. The edited cancer cells exhibit inhibited proliferation and suppressed migration and invasion. The therapeutic efficiency of genome editing in the cancer cell line as well as in CTCs can be facilely probed using the same targeting delivery vector to deliver molecular beacons into the living cells. The downregulated mRNA (CXCR4 mRNA) can be detected by a molecular beacon delivery system after the genome editing, and the upregulated mRNA (p53 mRNA and p21 mRNA) can be probed in situ by plasmid/molecular beacon co‐delivery systems. Through ex vivo studies on living CTCs in peripheral blood at the single‐cell resolution, this study provides a powerful and new platform to study the therapeutic responses of particular anti‐cancer treatments in cancer patients to provide accurate and timely information for personalized cancer therapy.

## Conflict of Interest

The authors declare no conflict of interest.

## Supporting information

Supporting Information

## Data Availability

The data that support the findings of this study are available from the corresponding author upon reasonable request.
