# Supplementary material for: Functional Tumor Targeting Nano‐Systems for Reprogramming Circulating Tumor Cells with In Situ Evaluation on Therapeutic Efficiency at the Single‐Cell Level
Source: Adv Sci (Weinh). 2022 May 20;9(21):2105806. doi: 10.1002/advs.202105806 (PMC9313495; doi:10.1002/advs.202105806)
Supplement: Supplementary file 1 — Supporting Information [file ADVS-9-2105806-s001.pdf]

Supporting Information

Functional Tumor Targeting Nano-Systems for Reprogramming Circulating Tumor Cells with *In Situ* Evaluation on Therapeutic Efficiency at the Single-Cell Level

*Xiao-He Ren, Xiao-Yan He, Chang Xu, Di Han, Si-Xue Cheng**

Materials and Methods

Materials

Histone was purchased from Sigma-Aldrich. Hyaluronic acid (HA) (sodium salt, $M_w = 5\times10^4$ Da) was supplied by Shandong Freda Biochem Co. Ltd (China). T22-NLS peptide (RRWCYRKCYKGYCYRKCR-PKKKRKV), and FITC labeled T22-NLS peptide were obtained from GL Biochem Co. Ltd (Shanghai, China). Lipofectamine 2000, YOYO-1 iodide, Hoechst 33342, and 3-(4,5-dimethylthiazol-2-yl)-2,5-diphenyltetrazolium bromide (MTT) were from Invitrogen. DAPI was from Solarbio.

An online guide design tool (http://crispr.mit.edu/) was used to design three sgRNA sequences (sgRNA$_1$: 5'-GAAGCGTGATGACAAAGAGG-3', sgRNA$_2$: 5'-ACGGCATCAACTGCCCAGAA-3', and sgRNA$_3$: 5'-CCCAAAGTACCAGTTTGCCA-3') for CXCR4 knockout (NCBI resource: CXCR4 Gene ID: 7852), and sgRNA$_3$ was identified as the most efficient one. The CRISPR-Cas9 plasmid for CXCR4 knockout containing sgRNA$_3$ (coded as “P”), and the negative control plasmid without sgRNA for CXCR4 knockout (with the negative control sequence of 5'-ACGGAGGCTAAGCGTCGCAA-3' instead of sgRNA) (coded as “CP”) were obtained from Genomeditech Co. Ltd. (Shanghai, China).

The locked nucleic acid molecular beacon for detection of CXCR4 mRNA coded as “MB1” (5'-Cy5-CGCACTCCTTGGCCTCTGACTGTTGGTGGTGCG-DABCYL-3', locked nucleic acids underlined, Genebank accession number: NM_003467), the molecular beacon for detection of p53 mRNA coded as “MB2” (5'-DABCYL-GTCGCAGCACAAACACGCACCTCAAAGCCTGCGACT-FAM-3', Genebank accession number: NM_000546) and the locked nucleic acid molecular beacon for detection of p21 mRNA coded as “MB3” (5'-Cy3-CGTCACCTGGGGACCCTTCAGCCTGACG-DABCYL-3', locked nucleic acids underlined, Genebank accession number: NM_000389) were from ShineGene Molecular Biotechnology Co. Ltd. (Shanghai, China).

Cell culture

HeLa cells and HEK293 cells were obtained from Procell Life Science & Technology Co. Ltd (Wuhan, China). Cells were cultured in Dulbecco's Modified Eagle's Medium (DMEM) containing 10% fetal bovine serum (FBS) (unless otherwise mentioned) and 100 U ml^{-1} antibiotics (penicillin−streptomycin) in a humidified 5% CO_2 atmosphere at 37 ºC.

Preparation of plasmid delivery systems

Histone (20 µg) in ultrapure water (50 µl) was gently mixed with CRISPR-Cas9 plasmid for CXCR4 knockout (2 µg) in ultrapure water (50 µl) for 10 min to form CRISPR-Cas9 plasmid@histone (coded as “P@H”) nanoparticles.

Histone (20 µg) and T22-NLS peptide (3 µg) in ultrapure water (50 µl) were gently mixed with CRISPR-Cas9 plasmid for CXCR4 knockout (2 µg) in ultrapure water (50 µl) for 10 min to form CRISPR-Cas9 plasmid@histone/peptide (coded as “P@HP”) nanoparticles.

Histone (20 µg) in ultrapure water (42.5 µl) was gently mixed with CRISPR-Cas9 plasmid for CXCR4 knockout (2 µg) in ultrapure water (50 µl) for 10 min. Then, hyaluronic acid (2 µg μl^{-1}, 7.5 µl) was added and gently mixed for 10 min to form CRISPR-Cas9 plasmid@histone/hyaluronic acid (coded as “P@HH”) nanoparticles.

Histone (20 µg) in ultrapure water (41 µl) was gently mixed with CRISPR-Cas9 plasmid for CXCR4 knockout (2 µg) in ultrapure water (50 µl) for 10 min to form CRISPR-Cas9 plasmid@histone complexes. T22-NLS peptide (2 µg μl^{-1}, 1.5 µl) was mixed with hyaluronic acid (2 µg μl^{-1}, 7.5 µl) to form peptide/hyaluronic acid complexes, and then added to CRISPR-Cas9 plasmid@histone complexes in ultrapure water (91 µl), followed by gentle mixing for 10 min to form CRISPR-Cas9 plasmid@histone/peptide/hyaluronic acid (coded as “P@HPH”) nanoparticles.

For comparison, nanoparticles loaded with the control plasmid (CP) without CXCR4 knockout function (control plasmid@histone/peptide/hyaluronic acid coded as “CP@HPH”), were prepared by similar procedures.

CRISPR-Cas9 plasmid@Lipofectamine 2000 complexes (P@Lip) and control plasmid@Lipofectamine 2000 complexes (CP@Lip) were prepared following the manufacturer protocols.

Preparation of molecular beacon delivery systems

Histone (20 µg) in ultrapure water (41 µl) was gently mixed with MB1 for detection of CXCR4 mRNA (0.1 nmol) in ultrapure water (50 µl) for 10 min to form MB1@histone complexes. T22-NLS peptide (2 µg μl^{-1}, 1.5 µl) was mixed with hyaluronic acid (2 µg μl^{-1}, 7.5 µl) to form peptide/hyaluronic acid complexes, and then added to MB1@histone complexes in ultrapure water (91 µl), followed by gentle mixing for 10 min to form MB1@histone/peptide/hyaluronic acid (coded as “MB1@HPH”) nanoparticles. MB2@histone/peptide/hyaluronic acid (MB2@HPH) nanoparticles and MB3@histone/peptide/hyaluronic acid (MB3@HPH) nanoparticles were prepared by similar procedures.

Preparation of plasmid/molecular beacon co-delivery systems

Histone (20 µg) in ultrapure water (41 µl) was gently mixed with CRISPR-Cas9 plasmid for CXCR4 knockout (2 µg) and the molecular beacon (MB2 for p53 mRNA detection, or MB3 for p21 mRNA detection) (0.1 nmol) in ultrapure water (50 µl) for 10 min to form CRISPR-Cas9 plasmid/MB2@histone complexes or CRISPR-Cas9 plasmid/MB3@histone complexes. T22-NLS peptide (2 µg µl^{-1}, 1.5 µl) was mixed with hyaluronic acid (2 µg µl^{-1}, 7.5 µl) to form peptide/hyaluronic acid complexes, and then added to CRISPR-Cas9 plasmid/MB2@histone complexes or CRISPR-Cas9 plasmid/MB3@histone complexes in ultrapure water (91 µl), followed by gentle mixing for 10 min to form CRISPR-Cas9 plasmid/MB2@histone/peptide/hyaluronic acid (coded as "P/MB2@HPH") nanoparticles or CRISPR-Cas9 plasmid/MB3@histone/peptide/hyaluronic acid (coded as "P/MB3@HPH") nanoparticles.

For comparison, control plasmid/MB2@histone/peptide/hyaluronic acid nanoparticles (CP/MB2@HPH), and control plasmid/MB3@histone/peptide/hyaluronic acid nanoparticles (CP/MB3@HPH) were prepared by similar procedures.

Characterizations of plasmid and/or molecular beacon delivery systems

The size and ζ potential of nanoparticles were measured by a Zetasizer (Nano ZS, Malvern Instruments). Before the measurement, 900 µl of ultrapure water was added in the ultrapure water containing nanoparticles (100 µl) for dilution.

To determine the encapsulation efficiency of plasmids and/or molecular beacons, 900 µl of ultrapure water was added in the ultrapure water containing nanoparticles (100 µl) for dilution, and then the sample was centrifuged at 10000 rpm for 1 h. The unencapsulated free plasmids and/or molecular beacons in the supernatants were measured using a Quant-iT PicoGreen dsDNA Assay Kit (Invitrogen) according to the manufacturer's protocol. Fluorescence spectroscopy (RF-5301 PC, Shimadzu) was used to determine the fluorescence intensity. The encapsulation efficiency of plasmids and/or molecular beacons was calculated as

$$\text{encapsulation efficiency} = (M_T - M_F) / W_T \times 100\%$$

where M_T was the total mass of plasmid and/or molecular beacon and M_F is the mass of unencapsulated free plasmid and/or molecular beacon.

To evaluate the assembly efficiency of T22-NLS peptide, FITC labeled T22-NLS was used to prepared the plasmid loaded nanoparticles. 900 µl of ultrapure water was added in the

ultrapure water containing nanoparticles (100 μl) for dilution, and then the sample was centrifuged at 10000 rpm for 1 h. The free FITC labeled peptide in the supernatant was measured with fluorescence spectroscopy (RF-5301 PC, Shimadzu). The results showed about 70% of FITC labeled T22-NLS was self-assembled onto CRISPR-Cas9 plasmid@histone/FITC labeled peptide/hyaluronic acid nanoparticles.

The nanoparticles supported on a 200-mesh copper grid and stained with phosphotungstic acid were observed by transmission electron microscopy (JEM-2100).

Cellular internalization study

CRISPR-Cas9 plasmid was labeled by YOYO-1, and the YOYO-1 labeled plasmid was used to prepared plasmid loaded nanoparticles. For flow cytometry analysis, cells were seeded in a 6-well plate (2×10^5 cells in 2 ml of DMEM per well). After incubation for 24 h, the culture medium was removed, and the fresh medium containing nanoparticles loaded with YOYO-1 labeled plasmid was added to the cells. After co-incubation with the plasmid loaded nanoparticles at a plasmid concentration of 2 μg ml^{-1} for 4 h, the cells were washed thrice by PBS, digested with trypsin, collected by centrifugation, resuspended in 200 μl of PBS, and analyzed by flow cytometry (Dakewe EXFLOW-206).

For confocal laser scanning microscopy (CLSM), cells were seeded in a glass-bottomed culture dish (35 mm) (1×10^5 cells in 1 ml of DMEM). After incubation at 37 °C for 24 h, the culture medium was removed, and the fresh medium containing nanoparticles loaded with YOYO-1 labeled plasmid was added to the cells. After co-incubation with the plasmid loaded nanoparticles at a plasmid concentration of 2 μg ml^{-1} for 4 h, the cells were washed thrice by PBS. The cell nuclei were stained by DAPI for 5 min, and then the cells were observed by CLSM at 400× magnification (PerkinElmer UltraVIEW VoX). To observe the endosomal escape ability, the cells were co-incubated with the nanoparticles loaded with YOYO-1 labeled plasmid for 2, 4, and 6 h, respectively, and then the cells were washed thrice by PBS, stained by LysoTracker Red (Invitrogen) for 45 min, and then stained by Hoechst 33342 for 15 min at 37 °C. After that, the cells were washed by PBS, and observed by CLSM at 600× magnification.

MTT assay

To evaluate cell viability, cells were seeded in a 96-well plate (5×10^3 cells in 100 μl DMEM per well). After incubation for 24 h, the medium was replaced by fresh medium containing plasmid loaded nanoparticles. After the cells were treated with plasmid loaded nanoparticles

at a plasmid concentration of 2 μg ml^{-1} for 48 h, MTT (5 μg μl^{-1}, 10 μl) was added in every well. After 4 h, the supernatant was removed, and 200 μl of DMSO was added in each well to dissolve the formazan crystals. The absorbance of the solution was measured at 570 nm by a microplate reader (Bio-Rad 550) to determine the OD value. The cell viability was calculated as

cell viability=$OD_{treated}$ /$OD_{control}$ ×100%

where $OD_{treated}$ was obtained from the cells treated by a particular agent and $OD_{control}$ was obtained from the untreated cells.

Western blot assay

The proteins in the unedited cells and genome edited cells were studied by Western blotting as detailed below. Cells were seeded in a 6-well plate (2×10^5 cells in 2 ml DMEM per well). After incubation for 24 h, the medium was replaced by fresh medium containing plasmid loaded nanoparticles, and the cells were treated with plasmid (CRISPR-Cas9 plasmid for genome editing, or control plasmid for comparison) loaded nanoparticles at a plasmid concentration of 2 μg ml^{-1} for 48 h. After that, the cells were washed with PBS triple times, lysed and suspended in sodium dodecyl sulfate (SDS) sample buffer containing 1% β-mercaptoethanol. Total protein extracts were subjected to SDS-polyacrylamide gel electrophoresis. After electrophoresis, the proteins were transferred to poly(vinylidene fluoride) membranes (Millipore). To block non-specific binding sites, the membranes were treated with TBST (Tris-buffered saline with Tween-20) containing 5% milk for 1 h. Then the membranes were incubated with the primary antibody overnight at 4 °C. After washing, the membranes were incubated with the secondary antibody for 1 h. Then an enhanced chemiluminescence system (Aspen) was used to detect the signals.

CXCR4 and CD44 in native HeLa and 293 cells were analyzed by Western blotting by using the cells without any treatment.

Quantitative polymerase chain reaction (qPCR) assay

The levels of CXCR4 mRNA, p53 mRNA, p21 mRNA, microRNA-21 and microRNA-221 in genome edited cells and unedited cells were analyzed by qPCR. Cells were seeded in a 6-well plate (2×10^5 cells in 2 ml DMEM per well). After incubation for 24 h, the medium was replaced by fresh medium containing plasmid (CRISPR-Cas9 plasmid for genome editing, or control plasmid for comparison) loaded nanoparticles, and the cells were treated with plasmid loaded nanoparticles at a plasmid concentration of 2 μg ml^{-1} for 48 h. After that, the cells were

collected and the total RNA was extracted with a High Pure RNA Isolation Kit (Invitrogen). An EntiLink™ 1st Strand cDNA Synthesis Kit (ELK Biotech) was used for the first cDNA strand synthesized, and then qPCR was performed on a StepOne Real-Time PCR system (Life Technologies) with EnTurbo™ SYBR Green PCR SuperMix (ELK Biotech). The relative RNA levels were measured by the $2^{-\Delta\Delta Ct}$ method.

T7 endonuclease I (T7E1) assay

Cells were seeded in a 6-well plate (2×10^5 cells in 2 ml DMEM per well). After incubation for 24 h, the medium was replaced by fresh medium containing plasmid loaded nanoparticles, and the cells were treated with CRISPR-Cas9 plasmid loaded nanoparticles at a plasmid concentration of 2 μg ml^{-1} for 48 h. After that, the genomic DNA was extracted from the cells using QuickExtract DNA extraction solution (Epicentre). Genomic regions of CXCR4 gene were amplified by PCR and then the homoduplex products of PCR were denatured, rehybridized under stepdown annealing conditions to generate homo- and heteroduplexes. The mixture of duplexes was digested with T7E1 (NEB) that can cleave heteroduplex DNA mismatched. The products were electrophoresed on a 2% agarose gel. For comparison, the extracted genomic DNA without denaturation and rehybridization was also treated by T7E1 to exclude false positive results.

DNA sequencing assay

Cells were seeded in a 6-well plate (2×10^5 cells in 2 ml DMEM per well). After incubation for 24 h, the medium was replaced by fresh medium containing P@HPH, and the cells were treated with P@HPH at a plasmid concentration of 2 μg ml^{-1} for 48 h. After that, the genomic DNA was extracted from the cells and amplified by PCR. The product of PCR was subjected to TA cloning. The colonies were sequenced using an 3730XL DNA analyzer (Applied Biosystems).

Analysis on CXCR4 labeled by antibody in cancer cells

Cells were seeded in a 6-well plate (2×10^5 cells in 2 ml of DMEM per well). After incubation for 24 h, the medium was replaced by fresh medium containing plasmid loaded nanoparticles. After the cells were treated with plasmid loaded nanoparticles at a plasmid concentration of 2 μg ml^{-1} for 48 h, the medium was removed. The cells were labeled by the anti-human PE conjugated CXCR4 antibody (200 μg ml^{-1}, 5 μl) (Biolegend) and then incubated on ice in the dark for 20 min, harvested and analyzed by flow cytometry. For comparison, untreated cells

were used as a control, and mouse IgG2a kappa isotype control (Biolegend) was also analyzed to exclude false positive cells.

Cell apoptosis assay

Cells were seeded in a 6-well plate (2×10^5 cells in 2 ml DMEM per well). After incubation for 24 h, the medium was replaced by fresh medium containing plasmid (CRISPR-Cas9 plasmid for genome editing, or control plasmid for comparison) loaded nanoparticles, and the cells were treated with plasmid loaded nanoparticles at a plasmid concentration of 2 μg ml^{-1} for 48 h. After that, the cells were collected and stained by an Annexin V-FITC/PI Staining Assay Kit (4A Biotech Co. Ltd., China) based on the manufacturer's protocol. The stained cells were analyzed by flow cytometry (Dakewe EXFLOW-206).

Wound healing assay

Cells were seeded in a 6-well plate (2×10^5 cells in 2 ml DMEM per well). After incubation for 24 h, the medium was replaced by fresh medium containing plasmid (CRISPR-Cas9 plasmid for genome editing, or control plasmid for comparison) loaded nanoparticles, and the cells were treated with plasmid loaded nanoparticles at a plasmid concentration of 2 μg ml^{-1} for 48 h. After that, the cells were collected and seeded in a new 6-well plate (1×10^5 cells in 2 ml DMEM per well). When the cells were grown to 90% confluence, a 200 µl micropipette tip sterile was used to scratch a straight line. Then the debris was removed by PBS washing, and fresh DMEM (2% FBS) was added in the plate. After incubation for 24 h, the cells were washed twice by PBS, and observed by an inverted microscope (Olympus IX73) at 200× magnification.

Transwell invasion assay

Cells were seeded in a 6-well plate (2×10^5 cells in 2 ml DMEM per well). After incubation for 24 h, the medium was replaced by fresh medium containing plasmid (CRISPR-Cas9 plasmid for genome editing, or control plasmid for comparison) loaded nanoparticles, and the cells were treated with plasmid loaded nanoparticles at a plasmid concentration of 2 μg ml^{-1} for 48 h. After that, the cells were suspended in 200 μl of serum-free DMEM and seeded into the Matrigel pro-coated upper chamber (1×10^4 cells per well in a 24-well chamber). 600 µl of DMEM (10% FBS) was added to the lower chamber to form a chemoattractant environment. After incubation for 24 h, the noninvasive cells remained on the upper chamber were removed by cotton swabs. The cells that invaded to the lower chamber were rinsed with PBS twice,

fixed with 4% paraformaldehyde for 20 min, and stained with 0.1% crystal violet for 10 min. Subsequently, the invading cells were visualized by an inverted microscope (Olympus IX73) at 200× magnification.

Detection on CXCR4 mRNA in edited and unedited cancer cells by molecular beacon delivery systems

Cells were seeded in a glass-bottomed culture dish (35 mm) (1×10^5 cells in 1 ml DMEM). After incubation for 24 h, the medium was replaced by fresh medium containing plasmid (CRISPR-Cas9 plasmid for genome editing, or control plasmid for comparison) loaded nanoparticles, and the cells were treated with plasmid loaded nanoparticles at a plasmid concentration of 2 μg ml^{-1} for 48 h. After that, MB1@HPH was added in the wells. After the cells co-incubated with MB1@HPH at a MB concentration of 0.1 nmol ml^{-1} for 4 h, the cells were washed thrice by PBS, stained by DAPI for 5 min and observed by CLSM (PerkinElmer UltraVIEW VoX) at 400× magnification.

Cells were seeded in a 6-well plate (2×10^5 cells in 2 ml DMEM per well). After incubation for 24 h, the medium was replaced by fresh medium containing plasmid (CRISPR-Cas9 plasmid for genome editing, or control plasmid for comparison) loaded nanoparticles, and the cells were treated with plasmid loaded nanoparticles at a plasmid concentration of 2 μg ml^{-1} for 48 h. After that, MB1@HPH was added in the wells. After the cells co-incubated with MB1@HPH at a MB concentration of 0.1 nmol ml^{-1} for 4 h, the cells were washed with PBS thrice, digested with trypsin, collected by centrifugation, suspended in 200 μl of PBS, and subsequently analyzed by flow cytometry (Dakewe EXFLOW-206).

Detection on p53 mRNA and p21 mRNA in edited and unedited cancer cells by plasmid/molecular beacon co-delivery systems

Cells were seeded in a glass-bottomed culture dish (35 mm) (1×10^5 cells in 1 ml DMEM). After incubation for 24 h, the medium was replaced by fresh medium containing plasmid and molecular beacon co-loaded nanoparticles (P/MB2@HPH, CP/MB2@HPH, P/MB3@HPH, and CP/MB3@HPH, respectively), and the cells were treated with plasmid and molecular beacon co-loaded nanoparticles at a plasmid concentration of 2 μg ml^{-1} and a MB concentration of 0.1 nmol ml^{-1} for 48 h. After that, the cells were washed thrice by PBS, stained by DAPI for 5 min and observed by CLSM (PerkinElmer UltraVIEW VoX) at 400× magnification.

Cells were seeded in a 6-well plate (2×10^5 cells in 2 ml DMEM per well). After incubation for 24 h, the medium was replaced by fresh medium containing plasmid and molecular beacon co-loaded nanoparticles (CP/MB2@HPH, P/MB2@HPH, CP/MB3@HPH, P/MB3@HPH, respectively), and the cells were treated with plasmid and molecular beacon co-loaded nanoparticles at a plasmid concentration of 2 μg ml^{-1} and a MB concentration of 0.1 nmol ml^{-1} for 48 h. After that, the cells were washed with PBS thrice, digested with trypsin, collected by centrifugation, suspended in 200 μl of PBS, and subsequently analyzed by flow cytometry (Dakewe EXFLOW-206).

Blood sample collection

Peripheral blood samples were obtained from cancer patients in Affiliated Hospital of Anhui Medical University. The study was approved by the Ethics Committee of Anhui Medical University (Approval number: 2021H001). Informed written consent of all participants was obtained, and all the experiments were conducted following pertinent guidelines.

Evaluation of the stability of the delivery system in whole blood

CRISPR-Cas9 plasmid was labeled by TOTO-3. T22-NLS peptide was labeled by FITC. The TOTO-3 labeled plasmid and FITC labeled T22-NLS were used to prepared plasmid loaded nanoparticles (P@HPH).

The EDTA-anticoagulated whole blood (4 ml) from the patient was placed in a 6-well plate (2 ml of whole blood per well). Then P@HPH loaded with 4 μg of CRISPR-Cas9 plasmid in 200 μl of ultrapure water were added to 2 ml of whole blood. After 4 h, the blood in each well was mixed with 50 ml of PBS for dilution, and then filtered with a 7 μm pore sized membrane filter to remove blood cells. CTCs on the filter membrane were fixed with 4% paraformaldehyde for 15 min, co-incubated with the anti-CXCR4 antibody (1:200 dilution) (Abcam) or anti-CK8/18/19 antibody (1:75 dilution) (Abcam) overnight, co-incubated with Cy3-labeled goat anti-rabbit IgG (H+L) (1:200 dilution) or Alexa Fluor 555-labeled donkey anti-mouse IgG (H+L) (1:200 dilution) (Beyotime), stained with DAPI for 5 min, and observed with CLSM under 1000× magnification.

Genome editing on CTCs by the cancer-targeting delivery system and detection on CXCR4 in genome edited CTCs as compared with unedited CTCs

The EDTA-anticoagulated whole blood (4 ml from each patient) was placed in a 6-well plate (2 ml of whole blood per well). Then P@HPH loaded with 4 μg of CRISPR-Cas9 plasmid in

200 μl of ultrapure water was added to 2 ml of whole blood in one well, and CP@HPH loaded with 4 μg of control plasmid in 200 μl of ultrapure water was added to 2 ml of whole blood in the other well. After 12 h, the blood in each well was mixed with 50 ml of PBS for dilution, and then filtered with a 7 µm pore sized membrane filter to remove blood cells. Subsequently, CTCs on the filter membrane were placed in a 6-well plate and incubated in DMEM (2 ml per well) for 36 h. Then the culture medium was removed and then the fresh DMEM containing MB1@HPH was added and CTCs was co-incubated with MB1@HPH at a MB concentration of 0.1 nmol ml^{-1} for 4 h. After that, the culture medium was removed. CTCs on the filter membrane were fixed with 4% paraformaldehyde for 15 min, co-incubated with anti-cytokeratin 8/18/19 (anti-CK8/18/19) (1:75 dilution) (Abcam) overnight, co-incubated with Alexa Fluor 555-labeled donkey anti-mouse IgG (H+L) (1:200 dilution) for 50 min (Beyotime), stained with DAPI for 5 min, and observed with CLSM under 1000× magnification.

For comparison, expression of CXCR4 protein in edited and unedited CTCs was detected by antibody labeling. CTCs were treated with P@HPH and CP@HPH (in whole blood for 12 h and then in DMEM for 36 h), respectively, as detailed above. After that, the culture medium was removed, and CTCs on the filter membrane were fixed with 4% paraformaldehyde for 15 min, co-incubated with anti-CK8/18/19 (1:75 dilution) and the antibody of CXCR4 (anti-CXCR4) (1:200 dilution) (Abcam) overnight, co-incubated with the Alexa Fluor 555-labeled donkey anti-mouse IgG (H+L) (1:200 dilution) and Alexa Fluor 647-labeled goat anti-rabbit IgG (H+L) (1:200 dilution) for 50 min (Beyotime), stained with DAPI for 5 min and observed with CLSM under 1000× magnification.

Genome editing on CTCs and detection on p53 mRNA and p21 mRNA in genome edited CTCs as compared with unedited CTCs by cancer-targeting plasmid/molecular beacon co-delivery systems

The EDTA-anticoagulated whole blood (4 ml from each patient) was placed in a 6-well plate (2 ml of whole blood per well). Then P/MB2@HPH or P/MB3@HPH nanoparticles loaded with 4 μg of CRISPR-Cas9 plasmid and 0.2 nmol of MB in 200 μl of ultrapure water were added to 2 ml of whole blood in one well, and CP/MB2@HPH or CP/MB3@HPH nanoparticles loaded with 4 μg of control plasmid and 0.2 nmol of MB in 200 μl of ultrapure water were added to 2 ml of whole blood in the other well. After 12 h, the blood in each well was mixed with 50 ml of PBS for dilution, and then filtered with a 7 µm pore sized membrane filter to remove blood cells. Subsequently, CTCs on the filter membrane were placed in a 6-

well plate and incubated in DMEM (2 ml per well) for 36 h. After that, the culture medium was removed. CTCs on the filter membrane were fixed with 4% paraformaldehyde for 15 min, co-incubated with anti-CK8/18/19 antibody (1:75 dilution) (Abcam) and the anti-CXCR4 antibody (1:200 dilution) (Abcam) overnight, co-incubated with Alexa Fluor 555-labeled donkey anti-mouse IgG (H+L) (1:200 dilution) or CoraLite 488-labeled goat anti-mouse IgG (H+L) (1:100 dilution) (Proteintech), and Alexa Fluor 647-labeled goat anti-rabbit IgG (H+L) (1:200 dilution) for 50 min (Beyotime), stained with DAPI for 5 min, and observed with CLSM under 1000× magnification.

Evaluation on viability of CTCs after genome editing

CTCs were treated with P@HPH (in whole blood for 12 h and then in DMEM for 36 h). After that, the culture medium was removed, and CTCs were stained with Calcein AM (Yeasen, China) with a concentration of 151 μg ml^{-1}, and observed with an inverted microscope (Olympus IX73) at 600× magnification.

Statistical analysis

The measurements were performed in triplicate, and data are given as mean ± standard deviation (s.d.). Statistical analysis was performed with GraphPad Prism 8 software using one-way analysis of variance (ANOVA) with Tukey’s multiple comparison test or a Student’s t test. $P < 0.05$ was considered statistically significant.

Table S1. Clinicopathological information of cancer patients and CTC detection results.

Patient ID	Cancer type	Gender	Age	TNM staging	Tumor size	Metastasis site	Total number of CTCs in 4 ml blood	MFI of CTCs before and after genome editing
BC0	Breast cancer	Female	65	T1N0M0	5.5 cm×3.0 cm×1.5 cm	-	8	N/A. The sample used for the study on the stability of the delivery system
SCLC	Small cell lung carcinoma	Male	68	ED	2.0 cm×2.0 cm×2.0 cm	Lung	43	Before: 9056 ± 807, n=23<br>After: 3593 ± 603, n=20<br>(CXCR4 antibody)
BC1	Breast cancer	Female	67	T2N0M0	3 cm×2.2 cm×1.5 cm	-	13	Before: 12331 ± 1003, n=7<br>After: 4496 ± 447, n=6<br>(CXCR4 mRNA)
BC2	Breast cancer	Female	41	T2N1M0	2.8 cm×1.1 cm	Axillary lymph node	21	Before: 6072 ± 556, n=10<br>After: 12549 ± 1274, n=11<br>(p53 mRNA)
BC3	Breast cancer	Female	64	T1N0M0	2 cm×1.5 cm×1 cm	-	17	Before: 6597 ± 978, n=9<br>After: 13971 ± 2254, n=8<br>(p21 mRNA)
BC4	Breast cancer	Female	42	T2N1M0	5 cm×4 cm×2 cm	Axillary lymph node	25	N/A. The sample used for CTC viability study

Sample code	Composition
P@H	CRISPR-Cas9 plasmid@histone
P@HP	CRISPR-Cas9 plasmid@histone/peptide
P@HH	CRISPR-Cas9 plasmid@histone/hyaluronic acid
P@HPH	CRISPR-Cas9 plasmid@histone/peptide/hyaluronic acid
CP@HPH	control plasmid@histone/peptide/hyaluronic acid
MB1@HPH	MB1@histone/peptide/hyaluronic acid
MB2@HPH	MB2@histone/peptide/hyaluronic acid
MB3@HPH	MB3@histone/peptide/hyaluronic acid
P/MB2@HPH	CRISPR-Cas9 plasmid/MB2@histone/peptide/hyaluronic acid
CP/MB2@HPH	control plasmid/MB2@histone/peptide/hyaluronic acid
P/MB3@HPH	CRISPR-Cas9 plasmid/MB3@histone/peptide/hyaluronic acid
CP/MB3@HPH	control plasmid/MB3@histone/peptide/hyaluronic acid

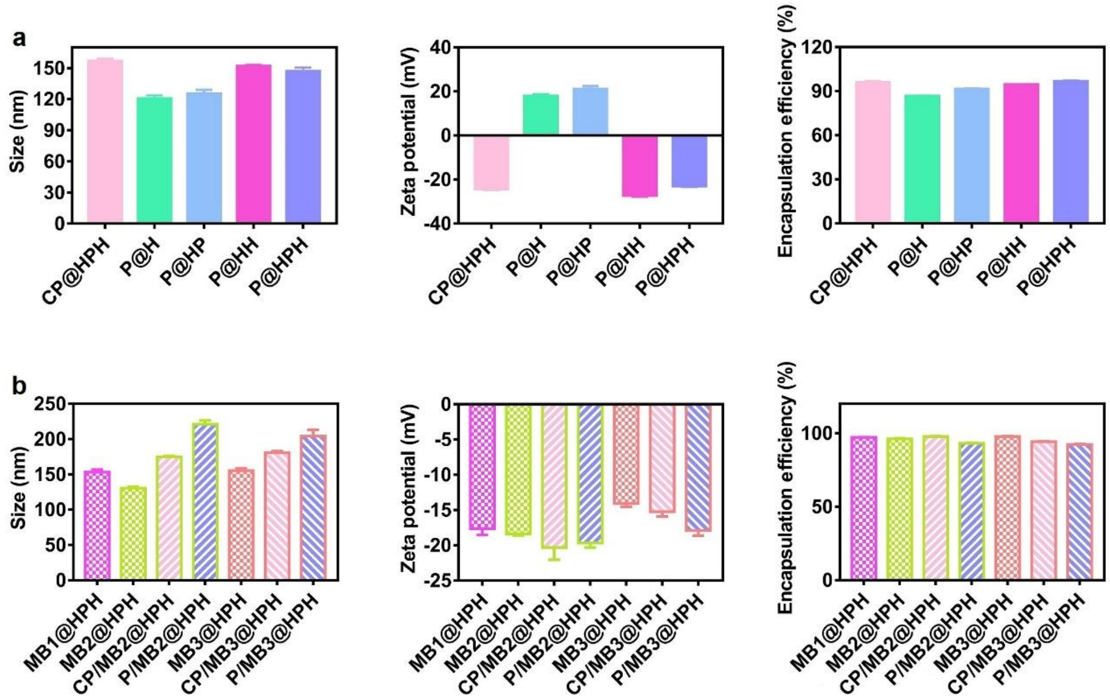

Figure S1. Compositions, particle sizes, zeta potentials, and plasmid and molecular beacon encapsulation efficiencies of delivery systems. (a) Plasmid delivery systems. (b) Molecular beacon delivery systems and plasmid/molecular beacon co-delivery systems. Data are mean ± s.d, n=3.

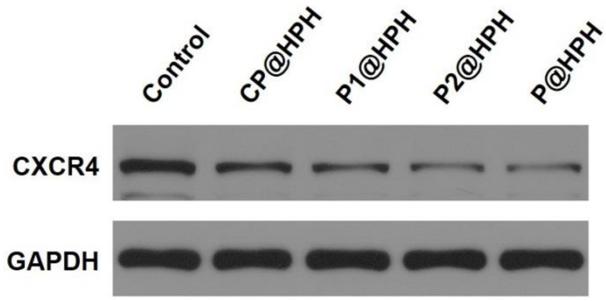

Figure S2. Western blot analysis on CXCR4 expression in cancer cells after gene editing by CRISPR-Cas9 plasmids with different sgRNA sequences (P1 with $sgRNA_1$, P2 with $sgRNA_2$, and P with $sgRNA_3$). HeLa cells were treated by tumor targeting delivery systems loaded with CRISPR-Cas9 plasmids or control plasmid for 48 h.

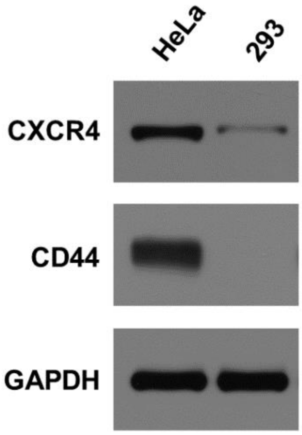

Figure S3. Western blot analysis on the expression of CXCR4 and CD44 in native HeLa and HEK293 cells.

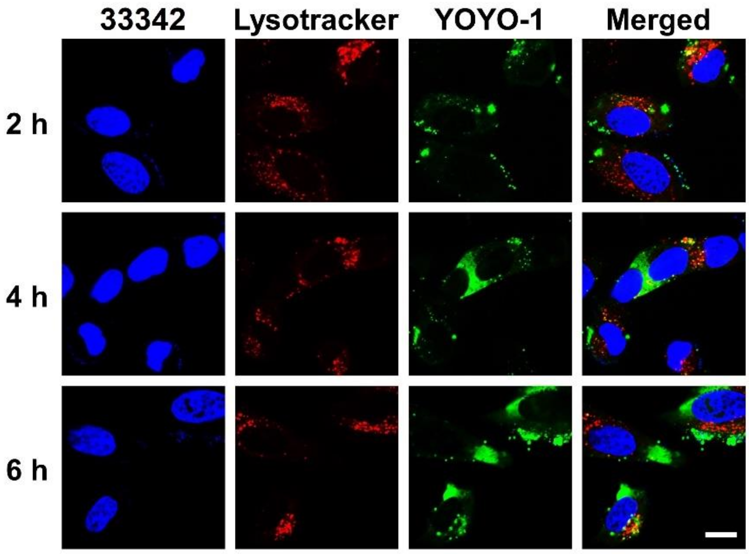

Figure S4. CLSM observation on endosomal/lysosomal escape of P@HPH. HeLa cells were co-incubated with P@HPH for 2 h, 4 h, and 6 h. CRISPR-Cas9 plasmid was labeled by YOYO-1 (green), nuclei were stained by Hoechst 33342 (blue) and endo/lysosomes were stained with LysoTracker (Red). Scale bar: 24 μm.

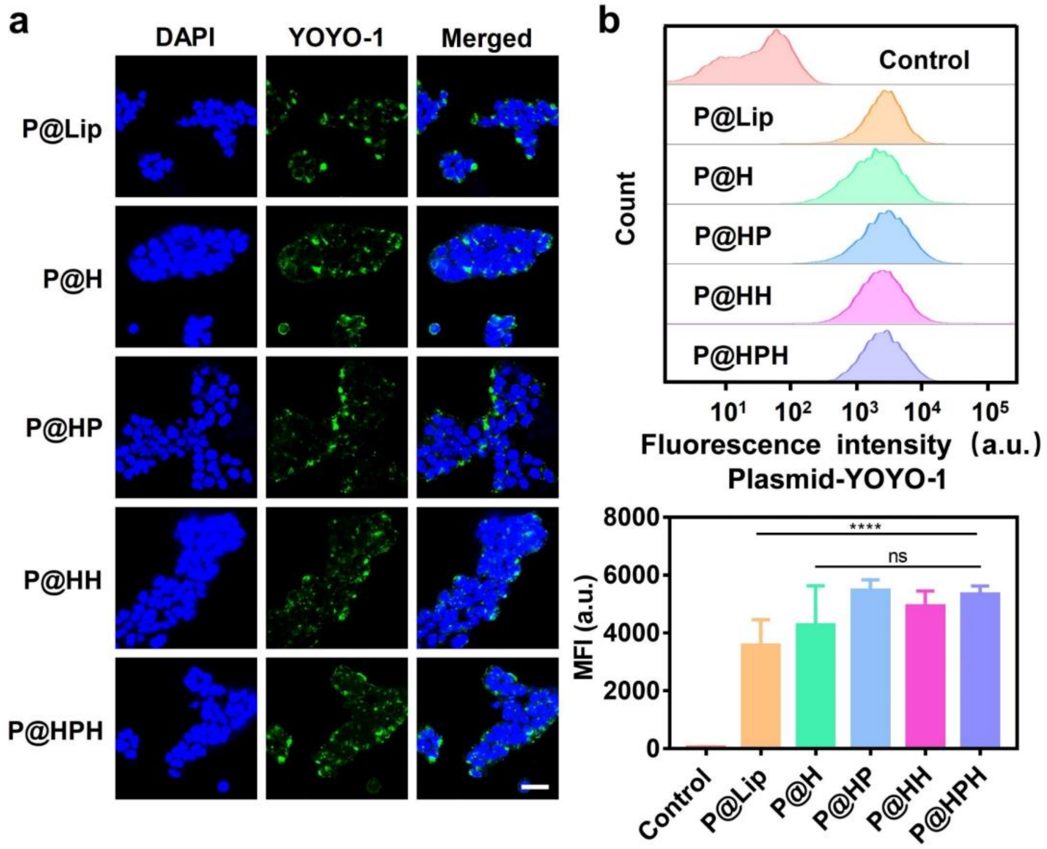

Figure S5. Study on cellular internalization of plasmid delivery systems in noncancer cells. (a) CLSM images of HEK293 cells treated by different plasmid delivery systems. (b) Flow cytometry analysis on HEK293 cells treated by different plasmid delivery systems. 293 cells were co-incubated with plasmid delivery systems for 4 h. Untreated 293 cells were served as a control. CRISPR-Cas9 plasmid was labeled by YOYO-1 (green), and nuclei were stained by DAPI (blue). Scale bar: 36 µm. Data are mean ± s.d, n=3. Statistical analysis was performed by using one-way ANOVA with Tukey's multiple comparison test. ****P< 0.0001.

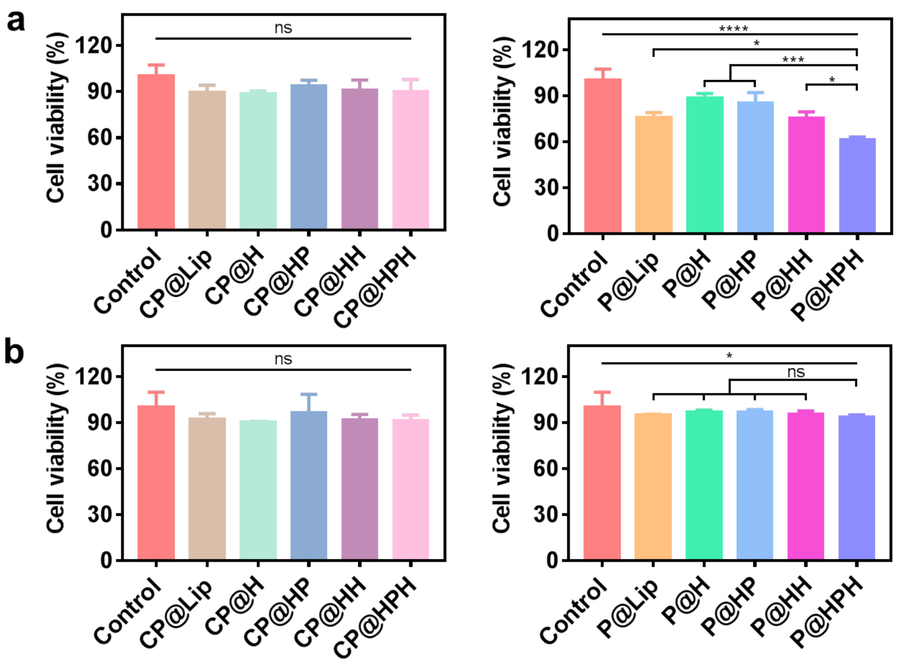

Figure S6. The cell viability after being treated with different plasmid delivery systems. (a) cancerous HeLa cells. (b) noncancerous HEK293 cells. The cells were treated with plasmid delivery systems for 48 h. Untreated cells were served as a control. Data are mean ± s.d, n=3. Statistical analysis was performed by using one-way ANOVA with Tukey's multiple comparison test. *P< 0.05, ***P< 0.001, ****P< 0.0001.

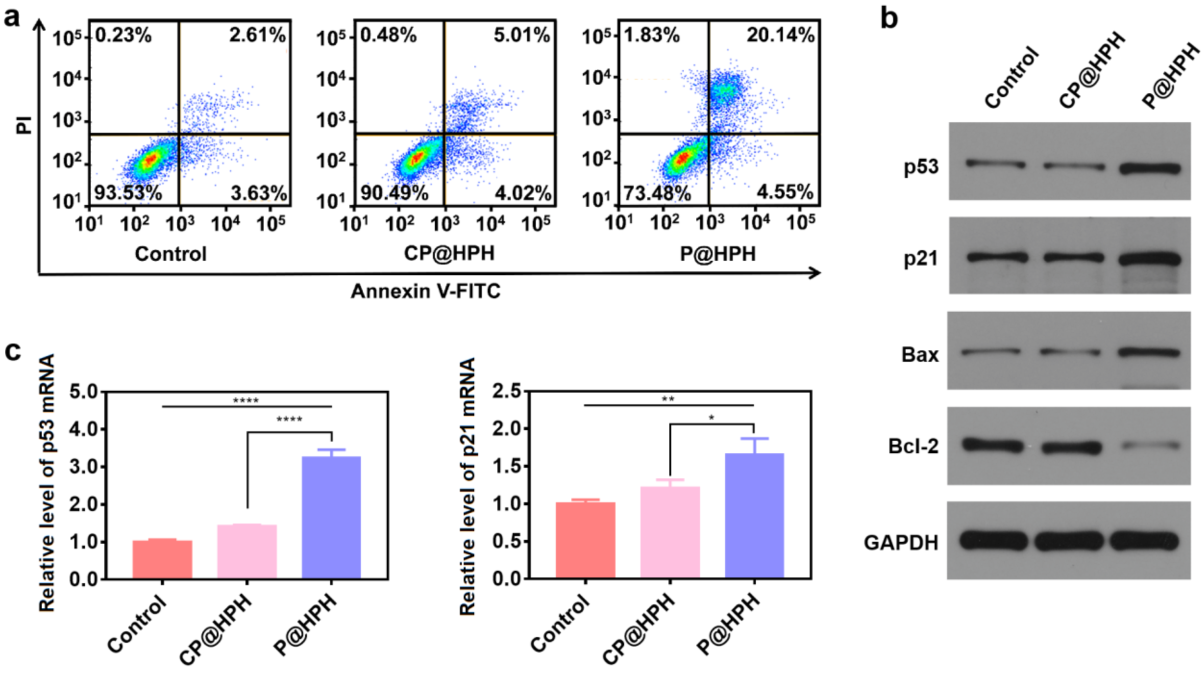

Figure S7. Effects of gene editing on cell apoptosis of cancer cells. (a) Flow cytometry analysis on cell apoptosis. (b) Western blot analysis on p53, p21, Bax and Bcl-2 expression. (c) qPCR analysis on p53 mRNA and p21 mRNA. HeLa cells were treated with CP@HPH or P@HPH for 48 h. Untreated cells were served as a control. Unedited cells treated by the control plasmid delivery system (CP@HPH) were studied for comparison. Data are mean ± s.d, n=3. Statistical analysis was performed by using one-way ANOVA with Tukey's multiple comparison test. *P< 0.05, **P< 0.01, ****P< 0.0001.

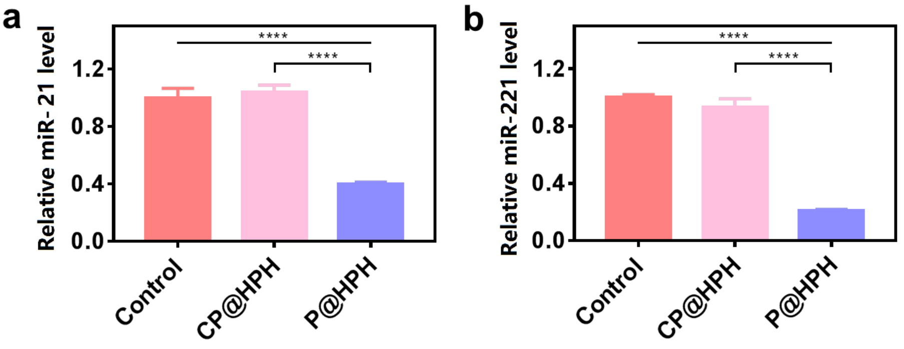

Figure S8. Effects of gene editing on microRNA levels in cancer cells. (a) Relative microRNA-21 (miR-21) level. (b) Relative microRNA-221 (miR-221) level. HeLa cells were treated with CP@HPH or P@HPH for 48 h. Untreated cells were served as a control. Unedited cells treated by the control plasmid delivery system (CP@HPH) were studied for comparison. Data are mean ± s.d, n=3. Statistical analysis was performed by using one-way ANOVA with Tukey's multiple comparison test. ****P< 0.0001.

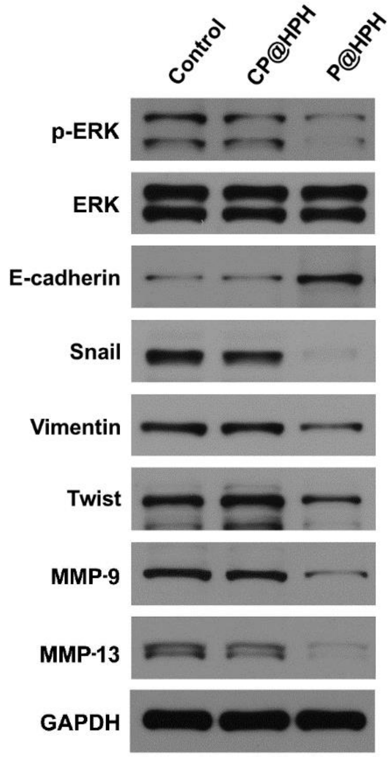

Figure S9. Effects of gene editing on expression of proteins involved in migration and invasion in cancer cells studied by Western blot analysis. HeLa cells were treated with CP@HPH or P@HPH for 48 h. Untreated cells were served as a control. Unedited cells treated by the control plasmid delivery system (CP@HPH) were studied for comparison.

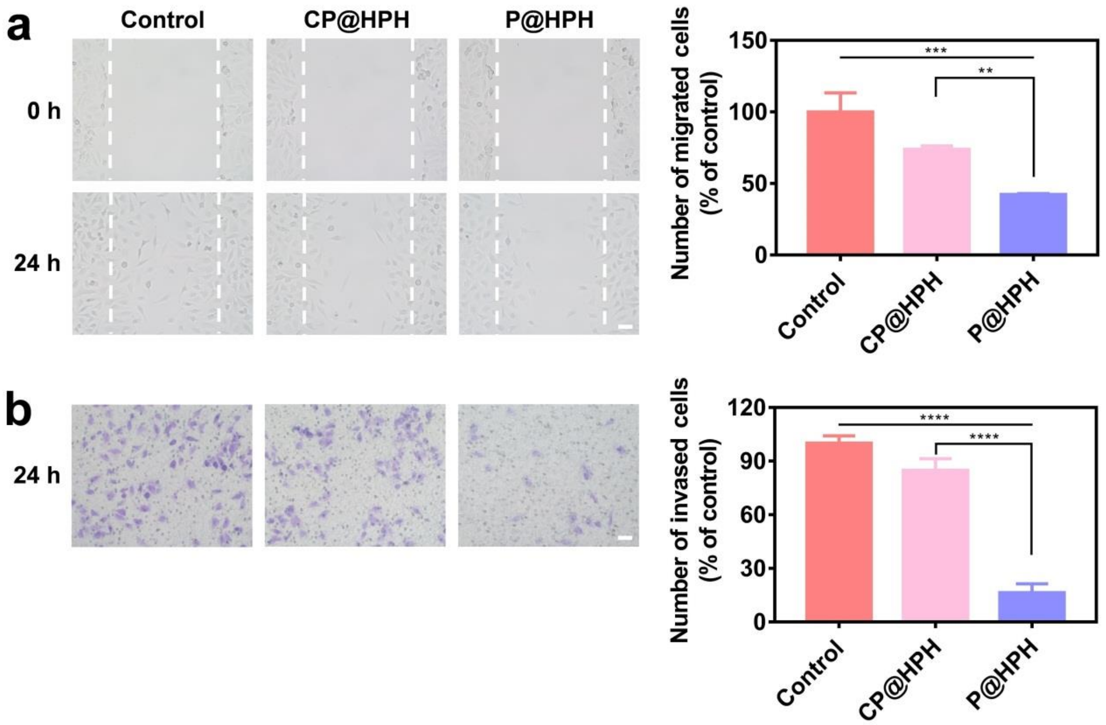

Figure S10. Effects of gene editing on migration and invasion of cancer cells. (a) Wound healing assay. Scale bar: 50 µm. (b) Trans-well invasion assay. Scale bar: 50 µm. HeLa cells were treated with CP@HPH or P@HPH for 48 h. Untreated cells were served as a control. Unedited cells treated by the control plasmid delivery system (CP@HPH) were studied for comparison. Data are mean ± s.d, n=3. Statistical analysis was performed by using one-way ANOVA with Tukey's multiple comparison test. **P< 0.01, ***P< 0.001, ****P< 0.0001.

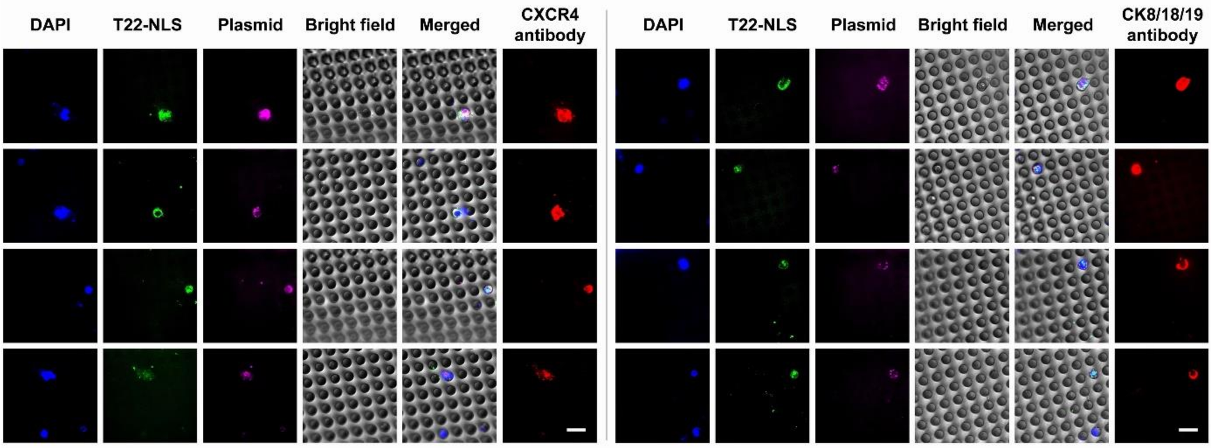

Figure S11. Study on the stability of plasmid delivery system in whole blood containing CTCs. P@HPH was added in the whole blood from the patient BC0 followed by co-incubation for 4 h. T22-NLS was labeled by FITC. CRISPR/Cas9 plasmid was labeled by TOTO-3. Cell nuclei were stained by DAPI. Scale bar: 15 µm.

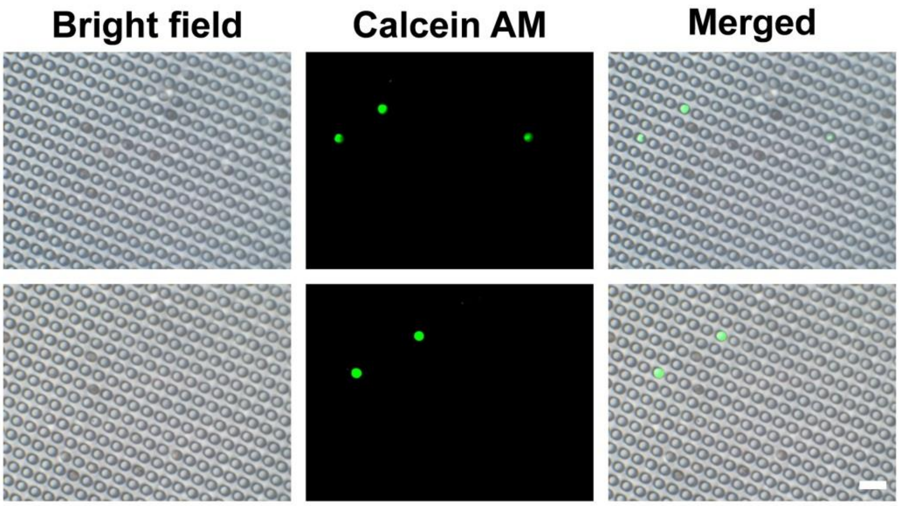

Figure S12. Calcein AM cell viability assay on the genome edited CTCs. CTCs from the patient BC4 were treated with P@HPH for 48 h, stained by calcein AM and observed by an inverted fluorescence microscopy. The two rows are different parts of the same sample. Scale bar: 20 µm.

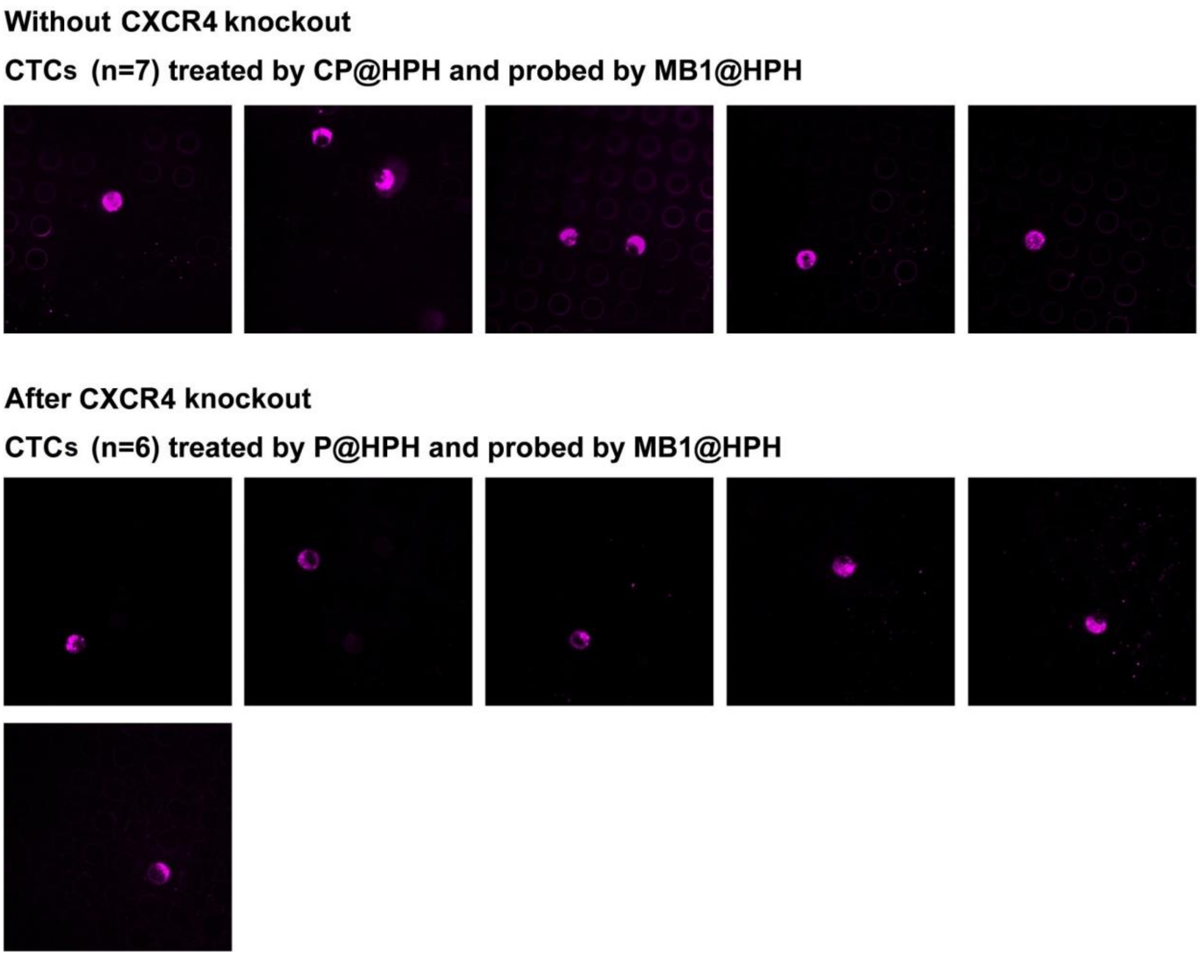

Figure S13. CXCR4 mRNA in unedited CTCs (without CXCR4 knockout) and edited CTCs (after CXCR4 knockout) as probed by the MB1 delivery system. CTCs from the patient BC1 were treated by plasmid delivery systems for 48 h and then treated by MB1@HPH for 4 h. CTCs were observed by CLSM at 1000× magnification.

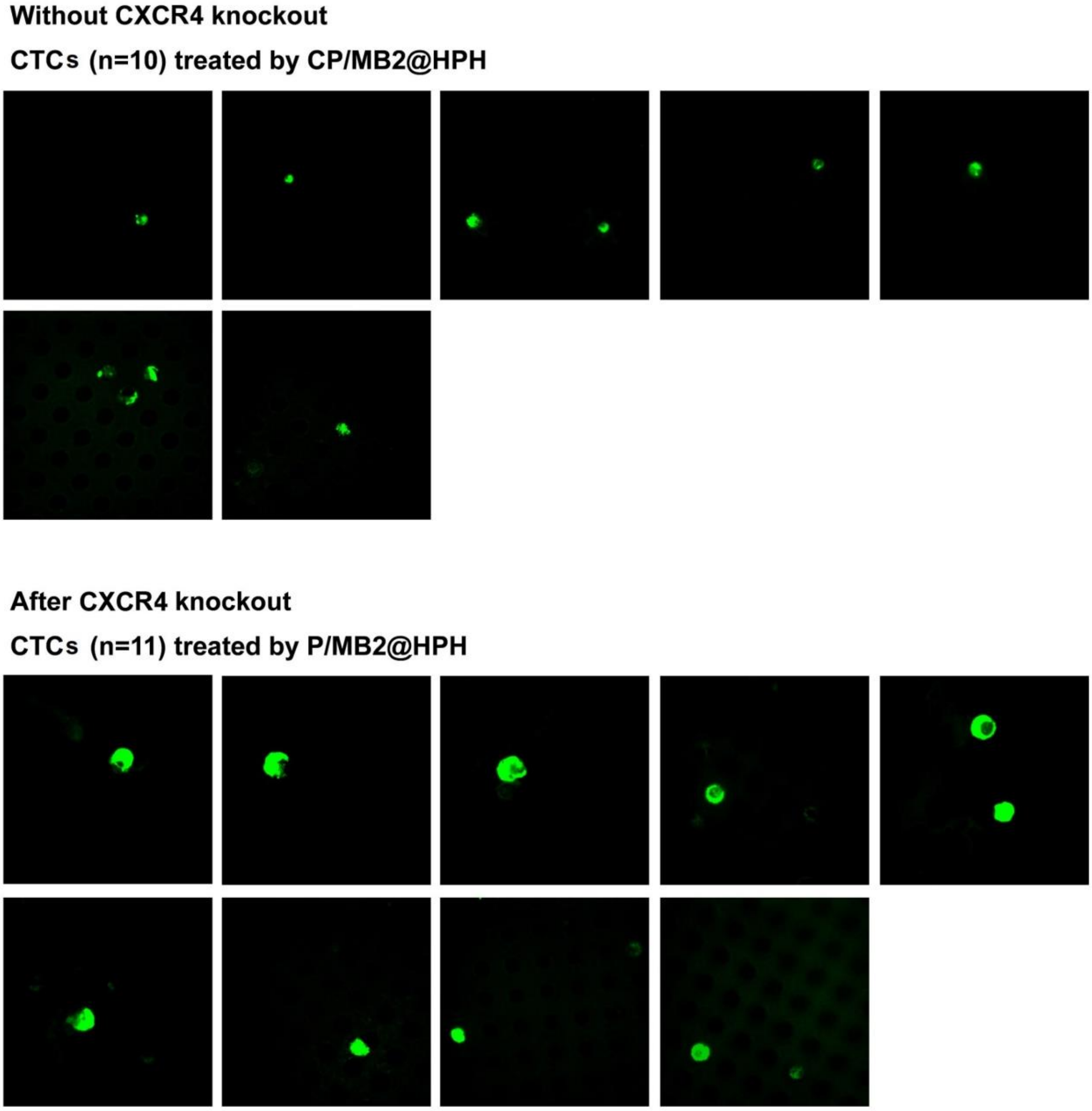

Figure S14. p53 mRNA in unedited CTCs (without CXCR4 knockout) and edited CTCs (after CXCR4 knockout) as probed by plasmid/MB2 co-delivery systems. CTCs from the patient BC2 were treated by plasmid/MB2 co-delivery systems for 48 h. CTCs were observed by CLSM at 1000× magnification.

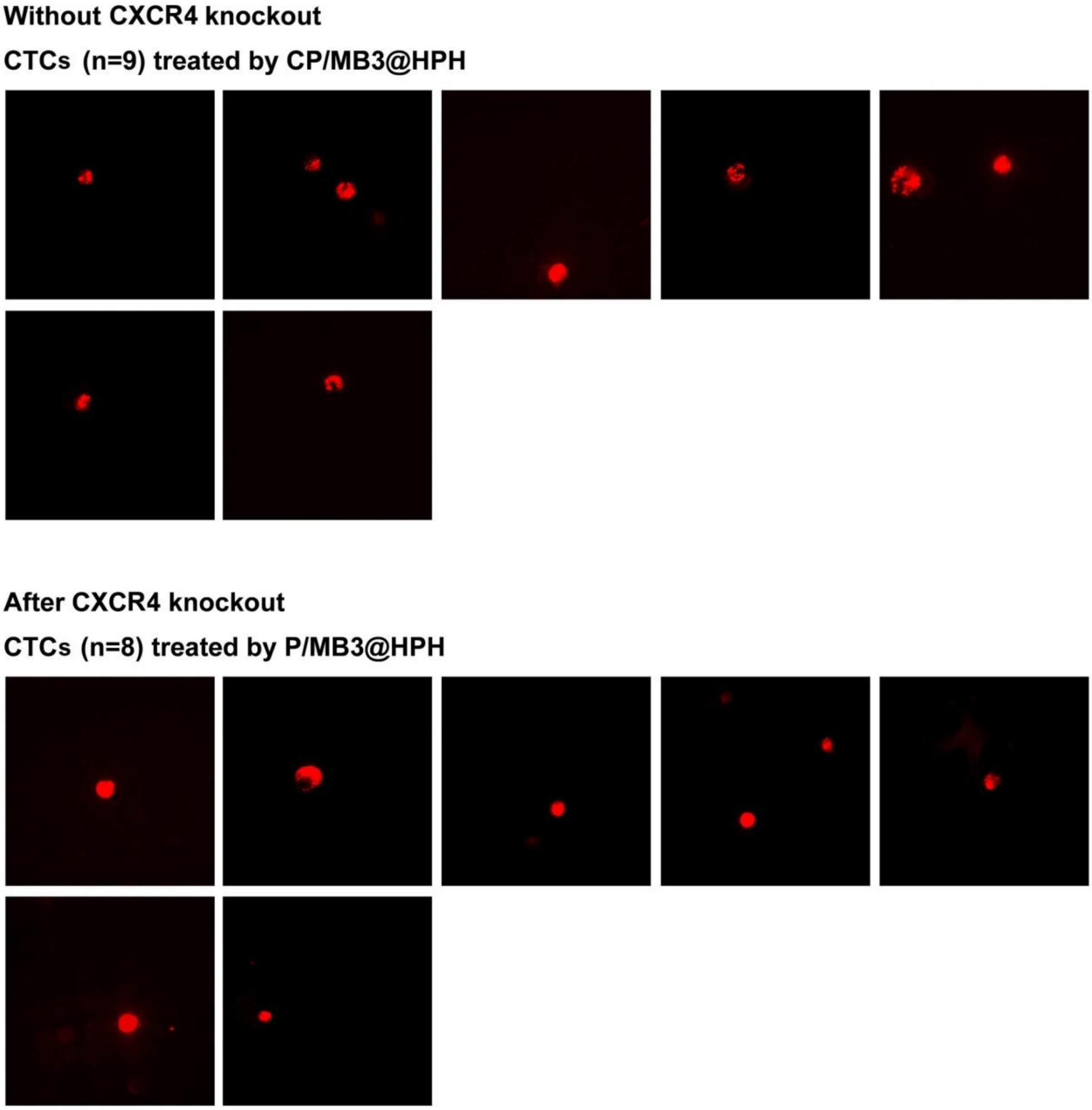

Figure S15. p21 mRNA in unedited CTCs (without CXCR4 knockout) and edited CTCs (after CXCR4 knockout) as probed by plasmid/MB3 co-delivery systems. CTCs from the patient BC3 were treated by plasmid/MB3 co-delivery systems for 48 h. CTCs were observed by CLSM at 1000× magnification.

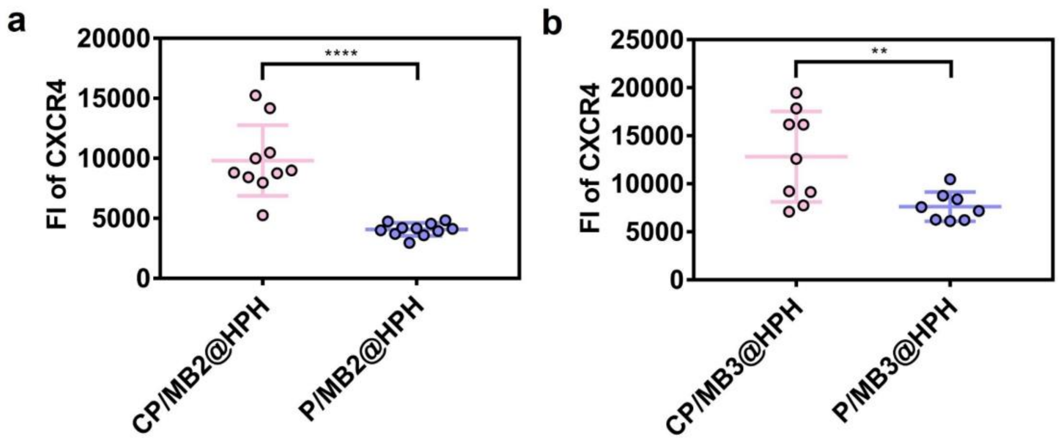

Figure S16. The CXCR4 protein levels of unedited CTCs and edited CTCs as determined antibody labeling. (a) CTCs from the patient BC2. CTCs were treated by CP/MB2@HPH (unedited) or P/MB2@HPH (edited) for 48 h before antibody labeling. (b) CTCs from the patient BC3. CTCs were treated by CP/MB3@HPH (unedited) or P/MB3@HPH (edited) for 48 h before antibody labeling. Fluorescence intensity of each CTC was analyzed by Volocity Demo 6.1.1 software. Statistical analysis was performed by using Student's t-test. $^{**}P<0.01$, $^{****}P<0.0001$.
